# Dual-Uptake Mode of the Antibiotic Phazolicin Prevents Resistance Acquisition by Gram-Negative Bacteria

**DOI:** 10.1128/mbio.00217-23

**Published:** 2023-02-21

**Authors:** Dmitrii Y. Travin, Romain Jouan, Armelle Vigouroux, Satomi Inaba-Inoue, Joy Lachat, Fazal Haq, Tatiana Timchenko, Dmitry Sutormin, Svetlana Dubiley, Konstantinos Beis, Solange Moréra, Konstantin Severinov, Peter Mergaert

**Affiliations:** a Center of Life Sciences, Skolkovo Institute of Science and Technology, Moscow, Russia; b Institute of Gene Biology, Russian Academy of Science, Moscow, Russia; c Université Paris-Saclay, CEA, CNRS, Institute for Integrative Biology of the Cell (I2BC), Gif-sur-Yvette, France; d Department of Life Sciences, Imperial College London, London, United Kingdom; e Rutherford Appleton Laboratory, Research Complex at Harwell, Didcot, United Kingdom; f Waksman Institute for Microbiology, Rutgers, Piscataway, New Jersey, USA; Case Western Reserve University School of Medicine

**Keywords:** phazolicin, antimicrobial peptides, ABC importers, SLiPT, substrate-binding protein, RiPP uptake

## Abstract

Phazolicin (PHZ) is a peptide antibiotic exhibiting narrow-spectrum activity against rhizobia closely related to its producer, *Rhizobium* sp. strain Pop5. Here, we show that the frequency of spontaneous PHZ-resistant mutants in Sinorhizobium meliloti is below the detection limit. We find that PHZ can enter S. meliloti cells through two distinct promiscuous peptide transporters, BacA and YejABEF, which belong to the SLiPT (SbmA-like peptide transporter) and ABC (ATP-binding cassette) transporter families, respectively. The dual-uptake mode explains the lack of observed resistance acquisition because the simultaneous inactivation of both transporters is necessary for resistance to PHZ. Since both BacA and YejABEF are essential for the development of functional symbiosis of S. meliloti with leguminous plants, the unlikely acquisition of PHZ resistance via the inactivation of these transporters is further disfavored. A whole-genome transposon sequencing screen did not reveal additional genes that can provide strong PHZ resistance when inactivated. However, it was found that the capsular polysaccharide KPS, the novel putative envelope polysaccharide PPP (PHZ-protecting polysaccharide), as well as the peptidoglycan layer jointly contribute to the sensitivity of S. meliloti to PHZ, most likely serving as barriers that reduce the amount of PHZ transported inside the cell.

## INTRODUCTION

Molecules of a peptidic nature constitute a considerable part of the known diversity of natural compounds with antimicrobial activity (1). The passage of intracellularly acting peptidic antibiotics through the cell envelope is a key step limiting their concentration inside the cell (2, 3). In Gram-negative bacteria, intracellularly acting peptidic antibiotics commonly cross the outer membrane via porins or TonB-ExbBD-dependent transporters and then rely on nonspecific peptide transporters for passage through the inner membrane (4, 5). Multiple antimicrobial peptides (AMPs) cross the inner membrane through peptide/H^+^ symporters similar to Escherichia coli SbmA (SLiPTs [SbmA-like peptide transporters]) (6). Several others cross the inner membrane via an unrelated ATP-binding cassette (ABC) importer, YejABEF, which is powered by ATP hydrolysis (4, 7, 8). Bacteria can pump out internalized peptidic antibiotics with the help of TolC-dependent efflux pumps, thus countering uptake and enhancing resistance (4, 5, 9).

**FIG 1:**
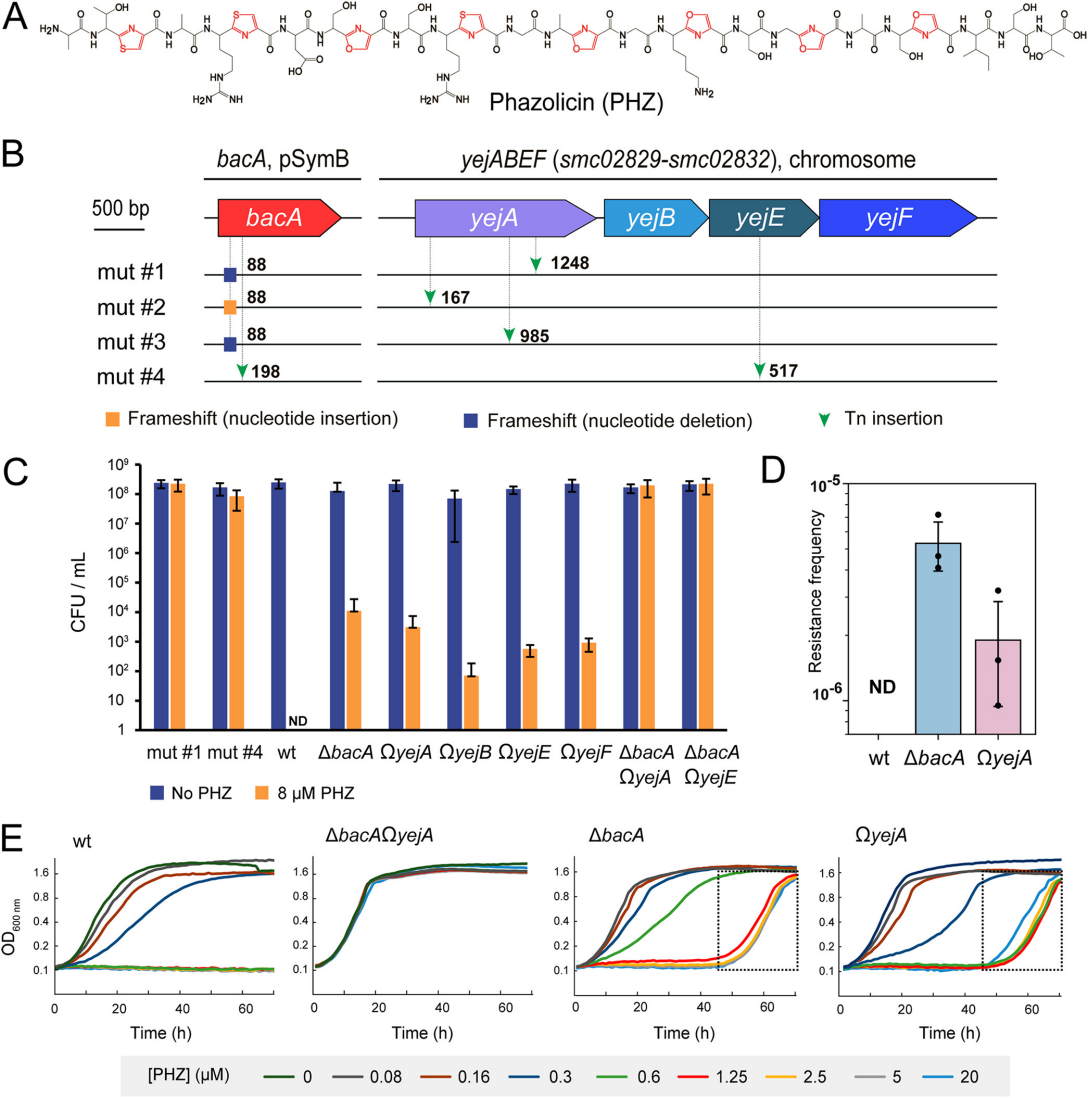
BacA and YejABEF transporters independently contribute to the uptake of phazolicin by Sinorhizobium meliloti. (A) Chemical structure of phazolicin. Posttranslationally installed thiazole and oxazole cycles are shown in red. (B) Schematic representation of mutations identified by whole-genome sequencing of four PHZ-resistant mutants of wild-type (wt) Sm1021. The numbers indicate the nucleotide positions in the gene where the mutation was detected. (C) CFU of Sm1021 growing on medium with (8 μM) and without PHZ. Results are presented as the averages from three biological replicates for the obtained PHZ-resistant strains (mutant 1 and mutant 4) and single and double mutants in the *bacA* and *yejABEF* genes. ND, not detected. Figure S1 in the supplemental material shows a picture of the CFU plating for one replicate. (D) Frequency of PHZ resistance acquisition in wt Sm1021 and *bacA* and *yejA* single-gene mutants. The data for three biological replicates are shown. (E) Growth curves of wt Sm1021, single mutants in the *bacA* and *yejA* genes, and the Δ*bacA* Ω*yejA* double mutant cultivated in the presence of PHZ at different concentrations. The parts of the growth curves corresponding to the delayed growth observed for single mutants in the presence of PHZ at concentrations above the MIC are shown in dotted rectangles.

Phazolicin (PHZ) is an azole-containing peptide produced by *Rhizobium* sp. strain Pop5 (10, 11). PHZ belongs to a class of natural products known as ribosomally synthesized and posttranslationally modified peptides (RiPPs) (12, 13). PHZ contains eight azole cycles installed into the structure of a precursor peptide through the cyclization of Ser and Cys residues by specific enzymes (Fig. 1A). PHZ is a translation inhibitor and prevents, at low-micromolar concentrations, the growth of bacteria from the *Rhizobium* and *Sinorhizobium* genera (10). Active export by the specific pump PhzE confers self-resistance to PHZ in the producing strain (10). Pop5 is a soil bacterium that establishes a symbiotic relationship with the legume Phaseolus vulgaris. It induces the formation of nodules, specific symbiotic root organs (14) that are colonized by nitrogen-fixing Pop5 bacteria. PHZ production may enable Pop5 to outcompete other rhizobia in bulk soil, during symbiosis, or both.

10.1128/mbio.00217-23.1 FIG S1 PHZ-resistant mutants. (A) CFU assay with 10-fold dilution series (dilution factors are indicated at the top) of wt Sm1021 and single and double mutants in *yejABEF* and *bacA* genes on YEB agar plates without PHZ and with 8 μM PHZ. Small black asterisks indicate occasional splashes of mutant 1 at the 0 and 10^−1^ dilutions. (B) Mutations identified by Sanger sequencing in the *bacA* gene of PHZ-resistant mutants selected using the Sm1021 Ω*yejA* strain. The numbers indicate the nucleotide positions in the gene, and the primers used for PCR amplification and sequencing are shown as blue arrows. (C) A fragment of the amino acid sequence alignment of BacA (S. meliloti) and SbmA (E. coli). (D) Structure of the E. coli SbmA dimer (subunits are shown in blue and light blue) (PDB accession no. 7P34 [6]). The amino acids homologous to those undergoing substitutions in PHZ-resistant mutants (clones B1 and B2) are shown as sticks and are in red. Download FIG S1, JPG file, 2.6 MB. Copyright © 2023 Travin et al. 2023 Travin et al. https://creativecommons.org/licenses/by/4.0/ This content is distributed under the terms of the Creative Commons Attribution 4.0 International license.

In this work, we apply genetic, biochemical, structural, and microbiological approaches to characterize the uptake of PHZ by Sinorhizobium meliloti. We identified two different mechanisms that determine the net sensitivity of S. meliloti to PHZ. Extracellular polysaccharides and the periplasmic peptidoglycan (PGN) form a barrier for PHZ uptake, while two inner membrane peptide transporters import the peptide. We further make use of transporter-deficient PHZ-resistant mutants to demonstrate that PHZ biosynthesis enables its producer to eliminate susceptible bacteria.

## RESULTS AND DISCUSSION

### PHZ-resistant S. meliloti mutants simultaneously carry mutations in genes of two peptide import systems.

To identify transporters mediating the internalization of phazolicin into the cells of PHZ-susceptible S. meliloti Sm1021 (referred to as Sm1021 here), we attempted to select spontaneous resistant mutants by plating 10^7^ cells onto solid medium containing 20 μM PHZ (approximately 20× MIC [10]). Since, unexpectedly, no resistant mutants were recovered, we constructed a transposon (Tn) mutant library of Sm1021 and repeated the screen with the same number of cells. This time, four resistant clones were obtained. To identify the mutations that they carried, the whole-genome sequence of each clone was determined. Transposon insertions were found in the *bacA*, *yejA*, and *yejE* genes (Fig. 1B). BacA is a SLiPT (6). YejA and YejE are subunits of the YejABEF transporter, which is composed of two transmembrane subunits (YejB and YejE), two nucleotide-binding subunits (YejF_2_), and a periplasmic substrate-binding protein (SBP) (YejA) (7). Note that mutants 1, 2, and 3, which had transposon insertions in *yejA*, also carried inactivating frameshifting point mutations at the beginning of *bacA*. In mutant 4, which carried a transposon insertion in *bacA*, the *yejE* gene was inactivated by another transposon insertion. Thus, in all PHZ-resistant mutants obtained, both the BacA and YejABEF inner membrane uptake systems are inactivated. This suggests that the simultaneous inactivation of both transporters is necessary for the acquisition of PHZ resistance and explains our initial failure to select spontaneous resistant mutants.

### BacA and YejABEF independently contribute to phazolicin uptake.

To confirm the role of both transporters in PHZ uptake, Δ*bacA* Ω*yejA* and Δ*bacA* Ω*yejE* double mutants of Sm1021 were constructed. Serial dilutions of double mutant cultures were spotted onto plates supplemented with PHZ along with the cultures of wild type (wt); mutants 1 and 4 (see above); Δ*bacA* (15), Ω*yejA*, Ω*yejB*, Ω*yejE*, and Ω*yejF* (7) single mutants, which were used as controls (Fig. 1C; see also Fig. S1A in the supplemental material). The *bacA* and all *yej* single mutants were susceptible to PHZ. In contrast, the Δ*bacA* Ω*yejA* and Δ*bacA* Ω*yejE* double mutants were fully resistant to PHZ, as were mutants 1 and 4. Together, these results demonstrate that the two transport systems independently contribute to PHZ import, that the presence of either one of them is sufficient for sensitivity, and that together they account for PHZ uptake by S. meliloti. No other transporters are involved.

We observed the formation of individual colonies in spots of undiluted or 10-fold-diluted cultures of single mutants (Fig. S1A). No such colonies were observed with the wt culture aliquots. The formation of PHZ-resistant colonies by the *bacA* and *yej* single mutants (Fig. S1A) as well as the outgrowth of these strains in liquid medium after a prolonged (50-h) incubation in the presence of concentrations above the MICs of PHZ (Fig. 1E) suggest that mutations leading to PHZ resistance spontaneously occur at an appreciable frequency in the single mutant backgrounds. We compared the frequencies of spontaneous resistance acquisition in the Δ*bacA* and the Ω*yejA* single mutants and wt Sm1021. As described above, no resistant clones were obtained for the wt. In contrast, for single mutants, PHZ-resistant colonies were recovered at a frequency of 10^−5^ to 10^−6^ (Fig. 1D). To understand the genetic basis of this resistance, we picked six random resistant clones of the Ω*yejA* strain (two from each biological replicate) and amplified their *bacA* genes. Sequencing revealed mutations in 5 out of 6 *bacA* amplicons (Fig. S1B). In three cases, single-nucleotide insertions or deletions led to premature stop codon formation in the *bacA* reading frame. Two other mutations led to single-amino-acid substitutions (L158R and F162S) in one of the BacA transmembrane α-helices. Since Leu158 and Phe162 face the inner part of the membrane (Fig. S1C and D), replacing these residues with charged or polar ones should inactivate the transporter. In the remaining mutant, no change in the *bacA* open reading frame was detected, and we speculate that a mutation in the promoter or another *bacA* regulatory element could inactivate BacA synthesis in this clone. Overall, these data confirm that mutations in *bacA* are the primary source of the acquisition of resistance by the strain with inactivated YejABEF. We surmise that a complementary result would be obtained with PHZ-resistant mutants selected in the Δ*bacA* background.

S. meliloti BacA was previously shown to internalize the thiazole-containing antibiotic bleomycin (BLM), a hybrid peptide-polyketide acting as a DNA-damaging agent (16). In E. coli, the BacA ortholog SbmA is required for the uptake of the azole-modified RiPPs microcin B17 (17) and klebsazolicin (18). Thus, PHZ provides another example of an azole-containing molecule internalized through a SLiPT. The exact mechanism of peptide recognition by SLiPTs is still unknown. Available SLiPT structures reveal a large cavity where structurally unrelated AMPs are thought to bind (6). The large size of the likely ligand-binding site explains the observed promiscuity of SLiPTs, which can also transport a number of other natural (19–22) and artificially designed (23) peptidic substrates.

In E. coli, YejABEF is the only point of entry for microcin C, a heptapeptidyl-nucleotide antibiotic targeting aspartyl-tRNA synthetase (8). Interestingly, when the peptide moiety of microcin C was artificially increased to 25 amino acids, import through SbmA also became possible, and both systems contributed to its internalization (24). These data are consistent with our observations of the uptake of the 27-amino-acid-long PHZ in Sm1021. Recently, YejBEF of Agrobacterium tumefaciens was demonstrated to be involved in peptidoglycan (PGN) recycling and to mediate the uptake of muropeptides generated by cell wall remodeling during growth (25). However, muropeptide uptake is performed in conjunction with the SBP YepA rather than with YejA, the cognate SBP of the transporter.

Strikingly, both BacA and YejABEF play critical roles in the symbiosis of S. meliloti with legume plants. Inside the legume nodules, these transporters are involved in the uptake of nodule-specific cysteine-rich peptides (NCRs) (7, 26), defensin-like AMPs produced by the host plant to control the endosymbiotic rhizobial population (27, 28). Since the internalization of NCRs by the two transporters protects the endosymbionts from their membrane-damaging activity, the *bacA* or *yejABEF* mutants form abnormally shaped endosymbiotic cells, many of which die (7, 29). Additionally, in pathogenic Salmonella, Brucella, and Mycobacterium strains, *yejABEF* and *bacA* or *sbmA* gene products protect the bacteria from host-produced membrane-targeting AMPs via peptide internalization (30–33). Note that in the case of membrane-attacking peptides such as the NCRs or the immunity AMPs of animals, BacA and YejABEF provide protection by peptide internalization, whereas for peptides with intracellular targets, such as PHZ, the activity of these transporters renders bacteria sensitive to the AMPs.

### PHZ-resistant Sm1021 avoids elimination by *Rhizobium* sp. Pop5 in coculture.

We performed coculture experiments with Pop5 and Sm1021 to test the hypothesis that PHZ synthesis by Pop5 restricts the growth of competing strains. To make sure that any observed effect of Pop5 on the growth of Sm1021 can be unambiguously linked to PHZ production, we used a PHZ-resistant Δ*bacA* Ω*yejA* Sm1021 mutant as a control and also constructed a non-PHZ-producing derivative of Pop5. This mutant, Pop5 Ω*phzD*, has a plasmid insertion in the *phzD* gene coding for the YcaO domain-containing cyclodehydratase required for posttranslational modifications of PHZ (10, 34) (Fig. S2A and B). Matrix-assisted laser desorption ionization–time of flight (MALDI-TOF) mass spectrometry confirmed that Pop5 Ω*phzD* was not producing mature PHZ (Fig. S2C). Consistently, no growth inhibition zones around the colonies of Pop5 Ω*phzD* spotted over a lawn of PHZ-susceptible Rhizobium leguminosarum 4292 were observed (Fig. S2D).

10.1128/mbio.00217-23.2 FIG S2 Construction and phenotype verification of the *phzD* mutant of *Rhizobium* sp. Pop5. (A) Biosynthetic gene cluster of PHZ (*phzEACBD*) in the genome of *Rhizobium* sp. Pop5. A region, internal to *phzD*, cloned into pVO155 for subsequent plasmid insertion mutagenesis is shown. Oligonucleotide primer pairs used for insertion verification are shown as blue and red arrows. (B) DNA gel electrophoresis of the PCR products amplified from the genomic DNA purified from two clones of the Ω*phzD* mutant and wt *Rhizobium* sp. Pop5. (C) Mass spectra of whole cells showing the loss of the prominent mass peak corresponding to mature phazolicin (2,363.9 [M + H]^+^) in the Ω*phzD* mutant in comparison to wt *Rhizobium* sp. Pop5. The induction of *phzD* gene expression from the pSRK plasmid leads to the restoration of PHZ production by the Ω*phzD* mutant. (D) The Rhizobium leguminosarum 4292 growth inhibition zone observed around the wt *Rhizobium* sp. Pop5 colony is not detected for the Ω*phzD* mutant. The phenotype of Pop5 Ω*phzD* experimentally confirms the association of PHZ with its biosynthetic gene cluster, which until now was based on the amino acid sequence of the encoded precursor peptide only. Download FIG S2, JPG file, 0.7 MB. Copyright © 2023 Travin et al. 2023 Travin et al. https://creativecommons.org/licenses/by/4.0/ This content is distributed under the terms of the Creative Commons Attribution 4.0 International license.

**FIG 2:**
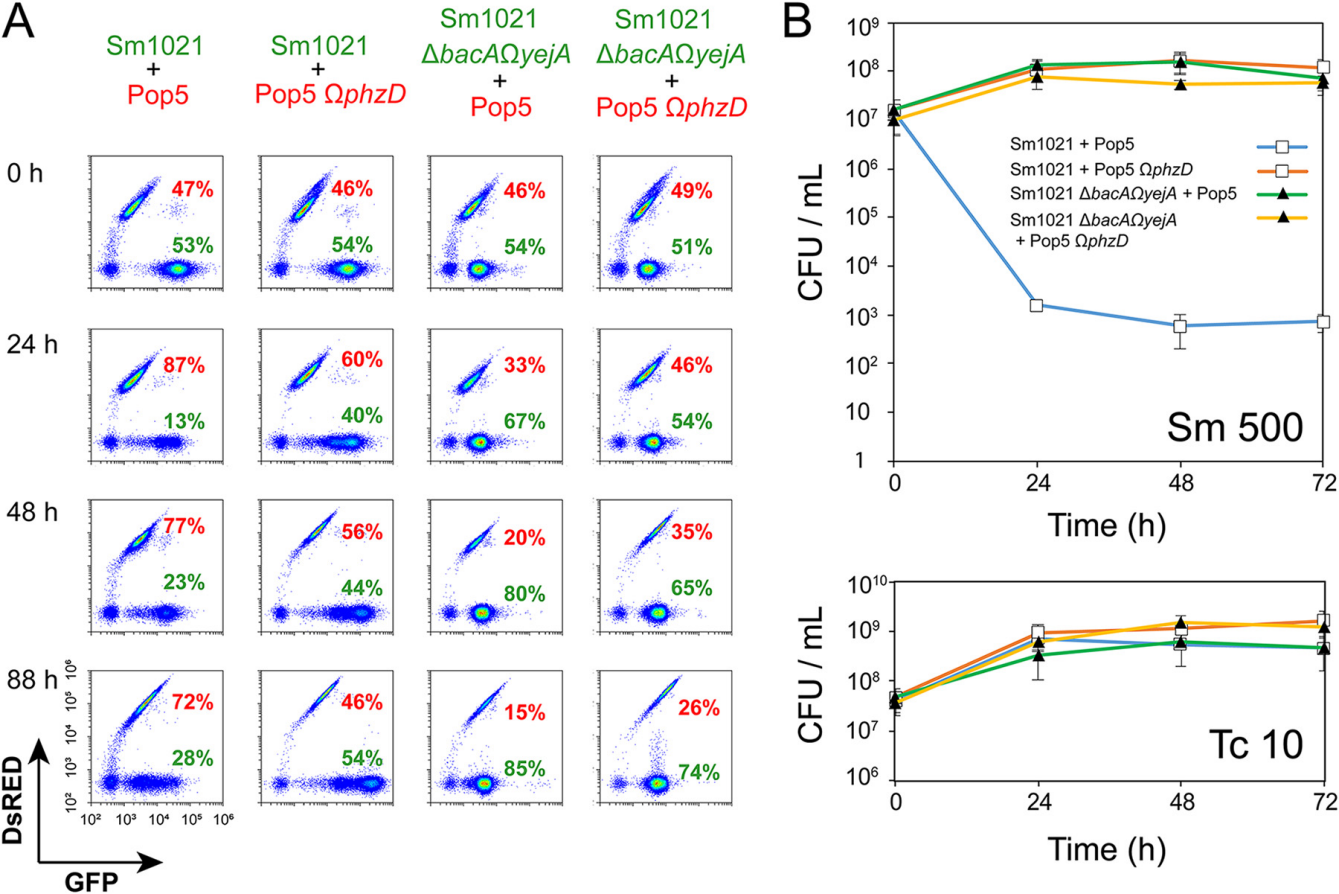
Sm1021 lacking both YejABEF and BacA avoids elimination by *Rhizobium* sp. Pop5 in cocultivation experiments. (A) Identification by flow cytometry of Sm1021 derivatives and Pop5 derivatives in cocultures grown for the indicated times. Dot plots show the GFP fluorescence on the *x* axis and DsRed fluorescence on the *y* axis for individual cells in the culture (dots). Note the higher GFP fluorescence of the Sm1021 strain carrying the pDG71 plasmid encoding GFP than that of the Sm1021 Δ*bacA* Ω*yejA* double mutant carrying a chromosome-inserted *gfp* copy. (B) CFU counts for the aliquots of two-strain mixtures sampled every 24 h and spotted onto petri dishes with selective media (growth on Sm 500 reflects the number of S. meliloti Sm1021 CFU with 500 μg · mL^−1^ Sm, and growth on Tc 10 reflects the number of *Rhizobium* sp. Pop5 CFU with 10 μg · mL^−1^ Tc).

We used flow cytometry to quantify green fluorescent protein (GFP)-marked S. meliloti derivatives (wt Sm1021 expressing GFP encoded on the plasmid pDG71 [35] and transporter-deficient Sm1021 Δ*bacA* Ω*yejA* expressing GFP from the plasmid insertion in the *yejA* gene) and DsRed-marked Pop5 derivatives (wt Pop5 and Pop5 Ω*phzD* expressing DsRed from plasmid pIN72) after cocultivation (Fig. 2A). The analysis at the onset of cocultivation (0 h) showed a roughly 1:1 ratio of *Rhizobium* to *Sinorhizobium* in the four tested Sm1021 derivative-Pop5 derivative mixtures. After growth for 24 h, 48 h, or 88 h, the proportion of wt Sm1021 cells was strongly reduced in a culture containing PHZ-producing wt Pop5. Moreover, the GFP fluorescence level of the Sm1021 cells remaining in cocultures was lower than that of cells in the initial mixture or in monoculture (Fig. S3A), indicating that GFP synthesis in Sm1021 is inhibited upon cocultivation with the PHZ producer, as expected. In other cocultivation combinations, the proportion of Sm1021 did not drop or even increased, and the GFP fluorescence of cells remained at initial levels.

10.1128/mbio.00217-23.3 FIG S3 Growth analysis of Sm1021 and Pop5 derivatives. (A) Identification by flow cytometry of individual strains grown in monocultures for the indicated times. Dot plots show the GFP fluorescence on the *x* axis and DsRed fluorescence on the *y* axis of individual cells in the culture (dots). (B) CFU counting for the pure cultures of Sm1021 (top) and Pop5 (bottom) derivatives on selective media containing Sm and Tc, respectively. No growth was observed for pure cultures of Sm1021 on Tc 10 and Pop5 on Sm 500. Download FIG S3, JPG file, 0.3 MB. Copyright © 2023 Travin et al. 2023 Travin et al. https://creativecommons.org/licenses/by/4.0/ This content is distributed under the terms of the Creative Commons Attribution 4.0 International license.

Although the flow cytometry analysis indicated that the proportion of wt Sm1021 cells dropped upon cocultivation with wt Pop5 cells, it remained relatively high (from 28 to 13%, depending on the time of cultivation). We repeated the cocultivation experiment using CFU counting on selective media to assess the number of viable cells of each strain in mixed cultures. wt Pop5 and Pop5 Ω*phzD* carrying pIN72 (tetracycline [Tc] resistant [Tc^r^]) were mixed in a 1:1 ratio with either wt Sm1021 or the Δ*bacA* Ω*yejA* mutant (both streptomycin [Sm] resistant [Sm^r^]) and cultivated for 4 days. In parallel, the growth of pure cultures of each strain was monitored. Culture aliquots were withdrawn at the start and every 24 h afterward and used for CFU counting on tetracycline- or streptomycin-containing medium (Fig. 2B and Fig. S3B). After 24 h of cocultivation, the number of CFU per milliliter of Sm1021 dropped by almost 4 orders of magnitude. No increase in the number of S. meliloti CFU per milliliter was observed afterward, while the number of Pop5 CFU per milliliter continued to increase. In contrast, for mixed cultures containing either transporter-deficient Sm1021 Δ*bacA* Ω*yejA* or non-PHZ-producing Pop5 Ω*phzD*, the numbers of CFU per milliliter on both selective media increased ~10-fold during the first 24 h of cultivation and remained stable afterward. We conclude that the transporter-deficient strain avoids the action of PHZ produced by Pop5, while the growth of PHZ-susceptible wt Sm1021 is efficiently inhibited. The differences in total cell counts (by flow cytometry) and viable cell counts (CFU) suggest that PHZ-affected bacterial cells persist in the coculture after the loss of viability.

The production of an unrelated antirhizobial RiPP, trifolitoxin, was shown to enhance the competitiveness of its producer, Rhizobium anhuiense bv. *trifolii* T24, in root colonization and the occupancy of nodules (36, 37). It is not yet known whether PHZ provides a competitive advantage to its producer either in soil or upon root nodulation. However, our cocultivation studies show that this is clearly the case under laboratory conditions. We identified biosynthetic gene clusters (BGCs) virtually identical to the *phz* BGC in multiple recently sequenced genomes of *Alphaproteobacteria* and one genome of a betaproteobacterium sampled around the globe (Fig. S4A). The occurrence of clusters guiding the biosynthesis of PHZ homologs (Fig. S4B and C) across bacteria of multiple genera (including both nodule-forming and plant-associated rhizosphere species) (Table S1) derived from geographically distant sampling sites indicates that the ability to produce PHZ-like compounds may be a common and efficient mechanism that provides bacteria with competitive advantages.

10.1128/mbio.00217-23.4 FIG S4 Biosynthetic gene clusters of PHZ-related compounds. (A) Sampling sites of the environmentally isolated strains with *phz*-like biosynthetic gene clusters in the genomes. (B) Schematic structures of the *phz*-like BGCs found across *Proteobacteria*. Variants of gene composition found in the genus *Phyllobacterium* (1) and other genera (2) are shown. The proposed functions of the encoded proteins are listed on the right. A multiple-sequence alignment of the amino acid sequences of the precursor peptides encoded by the *phz*-like BGCs is shown below. The alignment consensus is shown; color highlighting is based on the chemical properties of the amino acids and their conservation. Note the high degree of conservation for the residues converted into azole cycles and involved in PHZ binding with the ribosome across the precursors. (C) Maximum likelihood phylogenetic tree of PhzD (YcaO cyclodehydratase, the key enzyme for azole cycle installation) homologs, built using PhyML (S. Guindon, J.-F. Dufayard, V. Lefort, M. Anisimova, et al., Syst Biol 59:307–321, 2010, https://doi.org/10.1093/sysbio/syq010). The PhzD sequence from PHZ-producing *Rhizobium* sp. Pop5 is shown in boldface type. Download FIG S4, JPG file, 1.0 MB. Copyright © 2023 Travin et al. 2023 Travin et al. https://creativecommons.org/licenses/by/4.0/ This content is distributed under the terms of the Creative Commons Attribution 4.0 International license.

10.1128/mbio.00217-23.6 TABLE S1 Isolation sources for the strains with *phz*-like BGCs in the genome. Download Table S1, DOCX file, 0.02 MB. Copyright © 2023 Travin et al. 2023 Travin et al. https://creativecommons.org/licenses/by/4.0/ This content is distributed under the terms of the Creative Commons Attribution 4.0 International license.

### BacA and YejABEF homologs from other bacteria are capable of PHZ transport.

**FIG 3:**
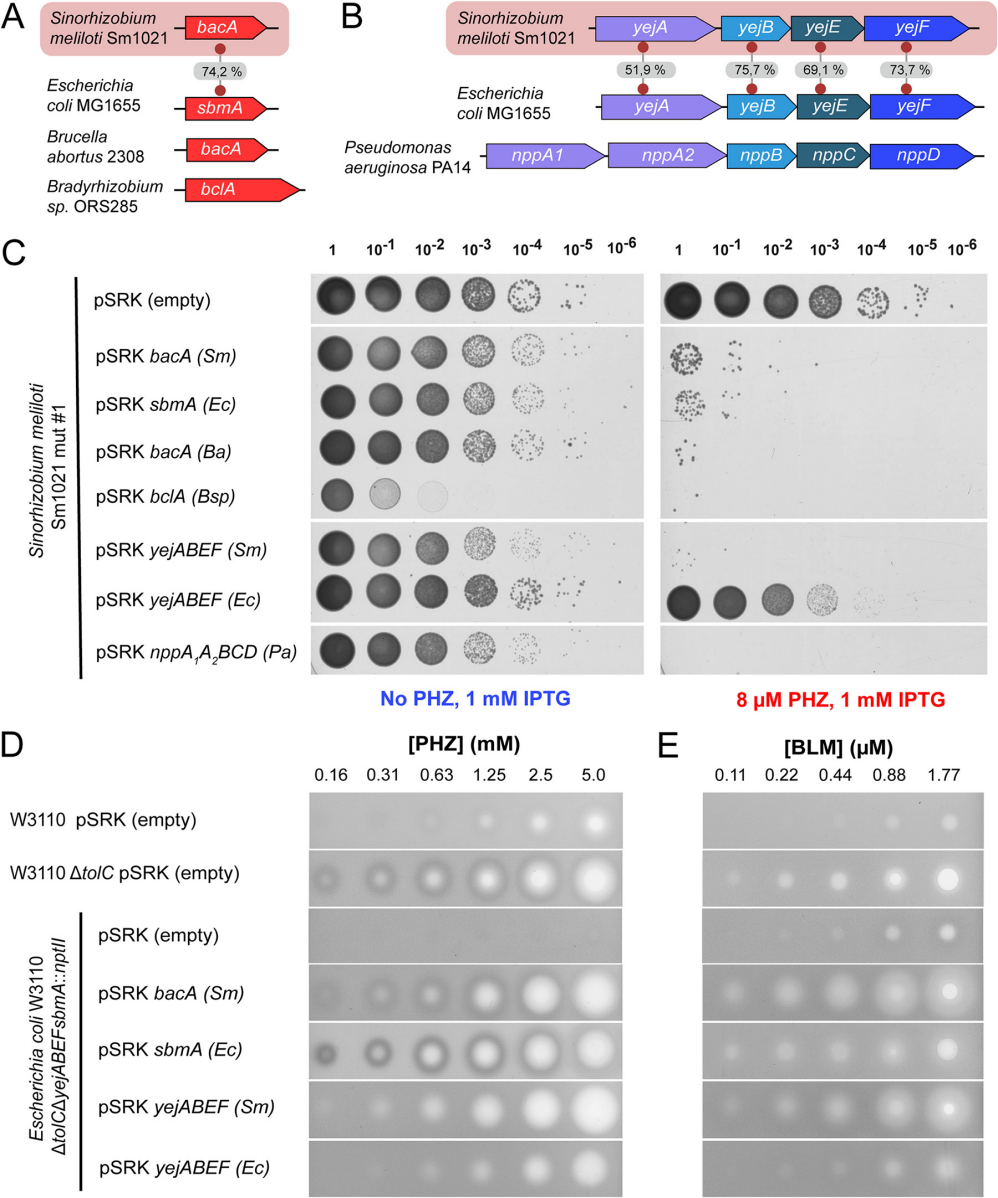
Episomal expression of *bacA*, *yejABEF*, and their orthologs from various *Alpha*- and *Gammaproteobacteria* promotes PHZ and BLM uptake. (A and B) Schematic representations of genes (*bacA*-like) and gene operons (*yejABEF*-like) that were chosen for expression in PHZ-resistant Sm1021 (mutant 1). Numbers on the gray background indicate the percent amino acid sequence similarity between the homologous proteins from S. meliloti Sm1021 and E. coli MG1655. (C) CFU assay with 10-fold dilution series (dilution factors are indicated at the top) on petri dishes with 8 μM PHZ and without PHZ for the strains of Sm1021 expressing different genes (operons) of transport proteins upon IPTG induction. Note that the CFU count for the *yejABEF*^Ec^-expressing strain (lane 9) is approximately 10 times lower in the presence of PHZ than in medium without PHZ, and this is not observed for the empty plasmid control (lane 1), indicating low PHZ uptake activity mediated by the pSRK *yejABEF*^Ec^ plasmid. *Sm*, S. meliloti; *Ec*, E. coli; *Ba*, Brucella abortus; *Bsp*, *Bradyrhizobium* sp. (D) Inhibition zones from 5 μL of PHZ spotted at the indicated concentrations onto lawns of E. coli derivatives grown on LB medium containing 1 mM IPTG. (E) Inhibition zones from 5 μL of BLM spotted at the indicated concentrations onto lawns of E. coli derivatives grown on LB medium containing 1 mM IPTG.

Genes encoding homologs of S. meliloti BacA (BacA^Sm^) and YejABEF^Sm^ are widely distributed across the *Alpha*- and *Gammaproteobacteria* (25). We constructed plasmids harboring several *bacA* and *yejABEF* homologs under the control of an inducible *lac* promoter. The selected transporters were previously shown to internalize peptide antibiotics (E. coli SbmA [SbmA^Ec^] and YejABEF^Ec^ [8] and NppA1A2BCD of Pseudomonas aeruginosa [38]) or to be required for the establishment of “host-symbiont” (BclA from *Bradyrhizobium* sp. [26]) or “host-pathogen” (Brucella abortus BacA [BacA^Ba^] [33]) relationships (Fig. 3A and B). These constructs, along with the empty vector control, were transformed into PHZ-resistant Sm1021 mutant 1 described above, and colony formation on plates with and without PHZ in the presence of 1 mM isopropyl-β-d-thiogalactopyranoside (IPTG) was monitored. With the exception of *yejABEF*^Ec^, strains expressing the genes of the selected transporters were susceptible to PHZ, while the strain harboring the empty vector was resistant (Fig. 3C). We conclude that SbmA^Ec^, NppA1A2BCD, BclA, and BacA^Ba^ can internalize PHZ.

The very moderate increase in PHZ sensitivity caused by YejABEF^Ec^ expression (Fig. 3C) may be due to the lower affinity of the periplasmic substrate-binding protein YejA^Ec^ for PHZ or may be caused by the poor assembly of the transporter in the heterologous host. To distinguish between these possibilities, we tested the ability of YejABEF^Ec^ to transport PHZ in its native host. E. coli is naturally highly resistant to PHZ (10). One factor contributing to this PHZ resistance is TolC (10), a major outer membrane multidrug efflux protein (9). A Δ*tolC* E. coli mutant has 8- to 16-fold-increased sensitivity to PHZ (Fig. 3D). Similarly, the MIC of PHZ for an Sm1021 Ω*tolC* mutant is four times lower than that for the wt (Table 1). To test the ability of peptide transporters to import PHZ in E. coli, we constructed a Δ*tolC* Δ*yejABEF sbmA*::*nptII* triple mutant; transformed it with *bacA*^Sm^, *sbmA*^Ec^, *yejABEF*^Sm^, or *yejABEF*^Ec^ expression plasmids; and determined PHZ sensitivity. Interestingly, the expression of either S. meliloti or E. coli transporters led to the appearance of inhibition zones, while the control strain was resistant to PHZ at the range of concentrations tested (Fig. 3D). Thus, YejABEF^Ec^ can transport PHZ. The lack of PHZ sensitivity upon the expression of *yejABEF*^Ec^ in Sm1021 is likely caused by inefficient expression or protein misfolding.

**TABLE 1:** Phazolicin and bleomycin MIC values for Sm1021 and its derivatives

Strain	Function or proposed function of the protein encoded by the disrupted gene	MIC (μg · mL^−1^) (MIC [μM])	
		PHZ	BLM
wt		1.47 (0.625)	0.25 (0.165)
Δ*bacA*	Inner membrane SLiPT	2.94 (1.25)	0.5 (0.33)
Ω*yejA*	Periplasmic substrate-binding protein of the YejABEF ABC transporter	1.47 (0.625)	0.5 (0.33)
Ω*yejE*	Transmembrane subunit of the YejABEF ABC transporter	1.47 (0.625)	0.5 (0.33)
Δ*bacA* Ω*yejA*	See above	>47 (>20)	8 (5.29)
Δ*bacA* Ω*yejE*	See above	>47 (>20)	8 (5.29)
Ω*tolC*	Outer membrane multidrug efflux protein	0.368 (0.15625)	<0.016 (<0.01)
Ω*smeA*	Membrane fusion protein of the SmeAB efflux pump	1.47 (0.625)	<0.016 (<0.01)
Ω*smb21252*	PPP biosynthesis (putative) glycosyltransferase	0.18 (0.078)	0.25 (0.165)
Ω*smb21265*	PPP biosynthesis (putative) glycosyltransferase family protein	0.18 (0.078)	0.25 (0.165)
Ω*rkpK*	Capsular polysaccharide biosynthesis, UDP-glucose 6-dehydrogenase	0.18 (0.078)	0.125 (0.08)
Ω*cyaA*	Adenylate cyclase	0.74 (0.3125)	0.25 (0.165)
Ω*smc00122*	PGN biosynthesis, putative penicillin-binding protein	2.94 (1.25)	0.25 (0.165)
Ω*relA*	GTP pyrophosphokinase, (p)ppGpp biosynthesis	2.94 (1.25)	0.25 (0.165)
Ω*phoR*	Phosphate regulon sensor histidine kinase	2.94 (1.25)	0.25 (0.165)

PHZ displays low-micromolar MICs against rhizobia closely related to the producing strain, while several other strains of rhizobia, E. coli, and other tested *Gammaproteobacteria* are resistant to high concentrations of PHZ (10). Since both SbmA^Ec^ and YejABEF^Ec^ are capable of enhancing PHZ sensitivity, and we previously demonstrated that E. coli ribosomes are highly sensitive to PHZ *in vitro* (10), it remains unclear why the PHZ MIC for E. coli is almost 3 orders of magnitude higher than that for S. meliloti. We propose that there may be additional factors contributing to the lower sensitivity of E. coli. TolC-mediated export cannot be the sole such factor since the E. coli Δ*tolC* mutant is still much more resistant to PHZ than wt S. meliloti. The levels of expression of genes coding for import and export machinery and differences in the specificities and kinetics of transport as well as other mechanisms (see below) may also contribute to the dramatic differences in MIC values. These observations demonstrate that the PHZ sensitivity of a strain is a complex phenotype that cannot be inferred solely from the presence of certain import and export systems.

### BacA and YejABEF are involved in the internalization of the PHZ-unrelated thiazole-containing antibiotic bleomycin.

Previously, experiments with the *bacA*-null mutant demonstrated that BacA^Sm^ contributes to the sensitivity of S. meliloti to BLM, but the incomplete resistance of the mutant pointed toward the involvement of an additional pathway in BLM internalization (16). We determined the MICs of BLM against wt Sm1021 and single and double *bacA* and *yej* mutants using broth microdilution assays (Table 1). In agreement with published data, compared to the wt, the Δ*bacA* mutant was two times less susceptible to BLM. Both the Ω*yejA* and Ω*yejE* single mutants had similarly increased resistance to BLM. The Δ*bacA* Ω*yejA* and Δ*bacA* Ω*yejE* double mutants were 16 times more resistant to BLM than the wt (Table 1). Thus, as is the case with PHZ, YejABEF and BacA provide independent pathways of BLM uptake. While these two transporters are largely responsible for the BLM sensitivity of Sm1021, the inhibition of the growth of the double mutants at high concentrations of BLM (Table 1) may be due to the function of yet another low-affinity transport system that remains to be identified or inefficient transporter-independent uptake.

BLM sensitivity was also not completely abolished by the simultaneous inactivation of the SbmA and YejABEF transporters in E. coli Δ*tolC* (Fig. 3E), suggesting an additional BLM uptake mechanism in E. coli as well. On the other hand, the expression of plasmid-borne *yejABEF*^Ec^ in the triple mutant did not enhance BLM sensitivity, contrary to *yejABEF*^Sm^. This suggests that unlike YejABEF^Sm^, YejABEF^Ec^ does not transport BLM.

### An *in vitro* transport assay shows the internalization of PHZ via BacA and SbmA.

**FIG 4:**
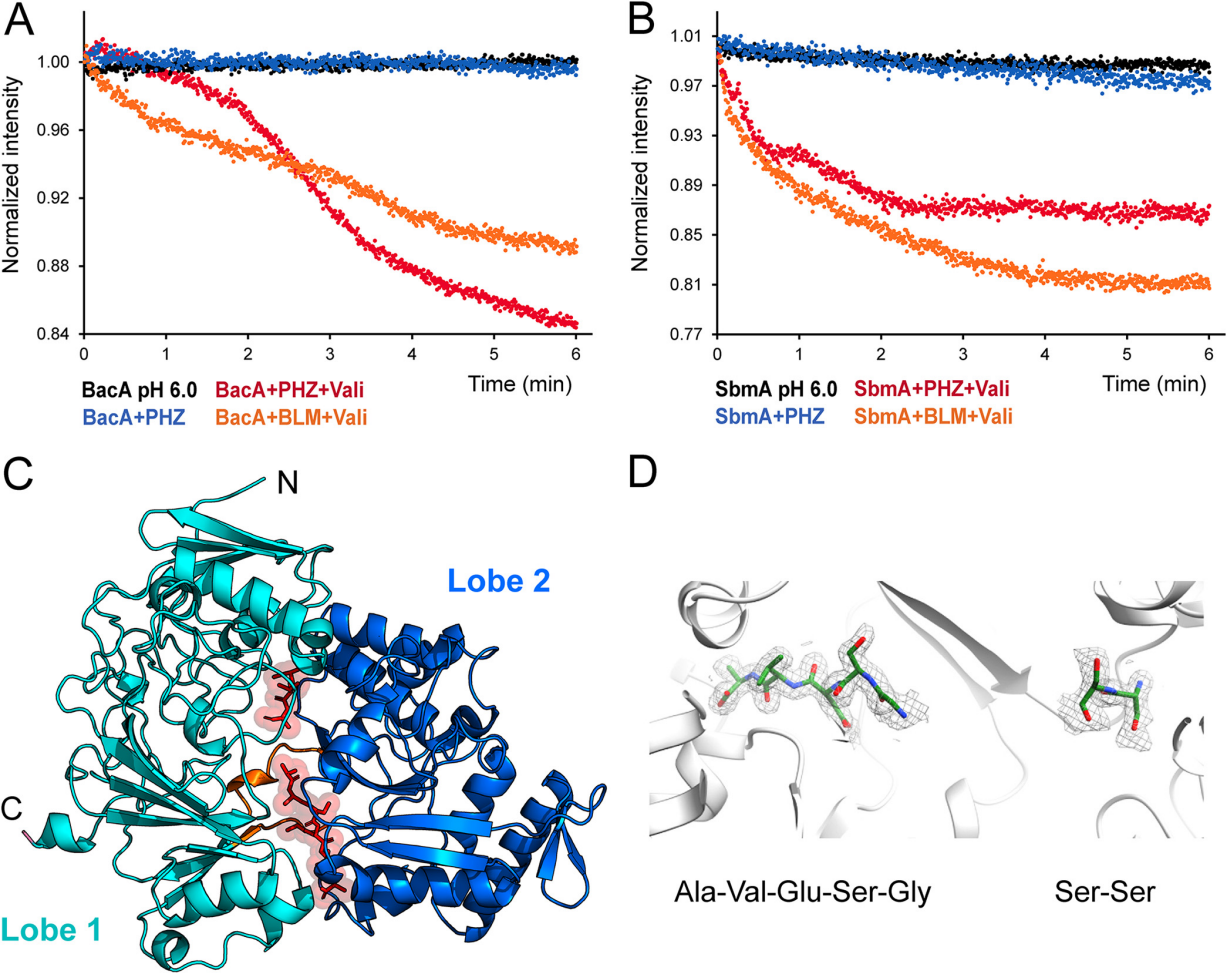
Biochemical characterization of BacA, SbmA, and YejA. (A and B) BacA (A) and SbmA (B) are involved in the symport of protons and PHZ inside proteoliposomes, determined by measuring the quenching of pyranine fluorescence upon liposome acidification. Bleomycin (BLM) was previously shown to be transported by both SbmA and BacA and was used as a positive control (6). Transport can be initiated only in the presence of valinomycin (Vali), which creates an efflux of potassium cations out of the liposomes, resulting in a charge gradient across the membrane. The tightness of the liposomes was assessed in the presence of external buffer (pH 6.0). For a description of the assay, see Materials and Methods. (C) Crystal structure of YejA^Sm^ with fortuitous peptides bound. Shown is a ribbon representation of YejA^Sm^ (PDB accession no. 7Z8E) with two distinct peptides (a pentapeptide and a dipeptide in stick format) in red, bound at the interface between the two YejA^Sm^ lobes (cyan and blue). The short hinge regions between the two lobes are shown in orange. (D) Electron density for the bound degraded peptides in the YejA^Sm^ binding pocket.

To provide further evidence that BacA^Sm^ and SbmA^Ec^ are directly involved in the transport of PHZ, we employed a liposome-based assay to monitor substrate transport (6). Both proteins belong to the SLiPT family that utilizes the proton gradient for the uptake of peptides, and the assay monitors the reduction of pyranine (trisodium 8-hydroxypyrene-1,3,6-trisulfonate) fluorescence inside liposomes upon the acidification of the lumen by proton-peptide symport by SLiPTs. The fluorescence decrease of pyranine inside liposomes containing BacA^Sm^ (Fig. 4A) or SbmA^Ec^ (Fig. 4B) is dependent on both a proton gradient artificially induced by valinomycin and PHZ. These results are consistent with the interchangeable roles of SbmA and BacA proteins and provide orthogonal evidence for the uptake of PHZ by SLiPTs, supporting our genetic observations. There is currently no *in vitro* system to study transport by YejABEF.

### Structure of YejA^Sm^.

To obtain insights into the mechanism for the recognition of PHZ by the YejABEF transporter, we sought to determine the structure of the substrate-binding protein YejA^Sm^ since in related transporters, the SBPs determine the specificity of transport (39, 40, 89). While we purified and crystallized the protein in the absence of externally added ligands, short peptides were found to be associated with YejA^Sm^. Our attempts to obtain YejA^Sm^ free of bound peptides were unsuccessful. The structure of YejA^Sm^ with bound peptides was determined at a 1.58-Å resolution. YejA^Sm^ adopts a fold consisting of two lobes, each formed by a central β-sheet flanked by α-helices connected with two short segments serving as hinges (Fig. 4C). The structure of YejA^Sm^ and the recently deposited structure of E. coli YejA (Protein Data Bank [PDB] accession no. 7ATR) share a highly similar cluster C fold within the SBP structural classification (41). The structure of YejA^Sm^ also resembles those of oligopeptide-binding SBPs such as MbnE from Methylocystis parvus (PDB accession no. 5ICQ) (42), AppA from Bacillus subtilis (PDB accession no. 1XOC) (43), and OppA from Lactococcus lactis (PDB accession no. 3DRF) (40). SBPs exist in either open (apo) or closed (ligand-bound) conformations (41). In our structure, YejA^Sm^ adopts a closed conformation due to the binding of fortuitous oligopeptides at the interface between the two lobes.

Although the YejA^Sm^ crystal structure corresponds to the full-length protein without signs of degradation, the bound oligopeptides could originate from YejA^Sm^ degradation and/or peptides from E. coli during overexpression. A dipeptide (SS) and a pentapeptide (GSDVA) were built, guided by the electron density at different places in the closed interface (Fig. 4D). Electron density linking the two peptides was missing, emphasizing that a population of different peptides might have bound to the peptide-binding site. Consequently, the resultant crystal structure of YejA^Sm^ is an average of different peptides in the crystal lattice. The binding of the pentapeptide to YejA^Sm^ is almost exclusively due to hydrogen bond formation between the peptide backbone and the protein (10 interactions out of 12), and that of the dipeptide is only via its backbone, meaning that the binding is unspecific. The presence of the peptides bound to YejA^Sm^ precluded us from solving the structure with PHZ. Presumably, the closed conformation of peptide-bound SBPs and the tight binding of the contaminating peptides selected from the pool available in the cytoplasm did not allow their exchange with an externally added antibiotic.

### Genome-wide identification of genetic determinants of PHZ sensitivity.

**FIG 5:**
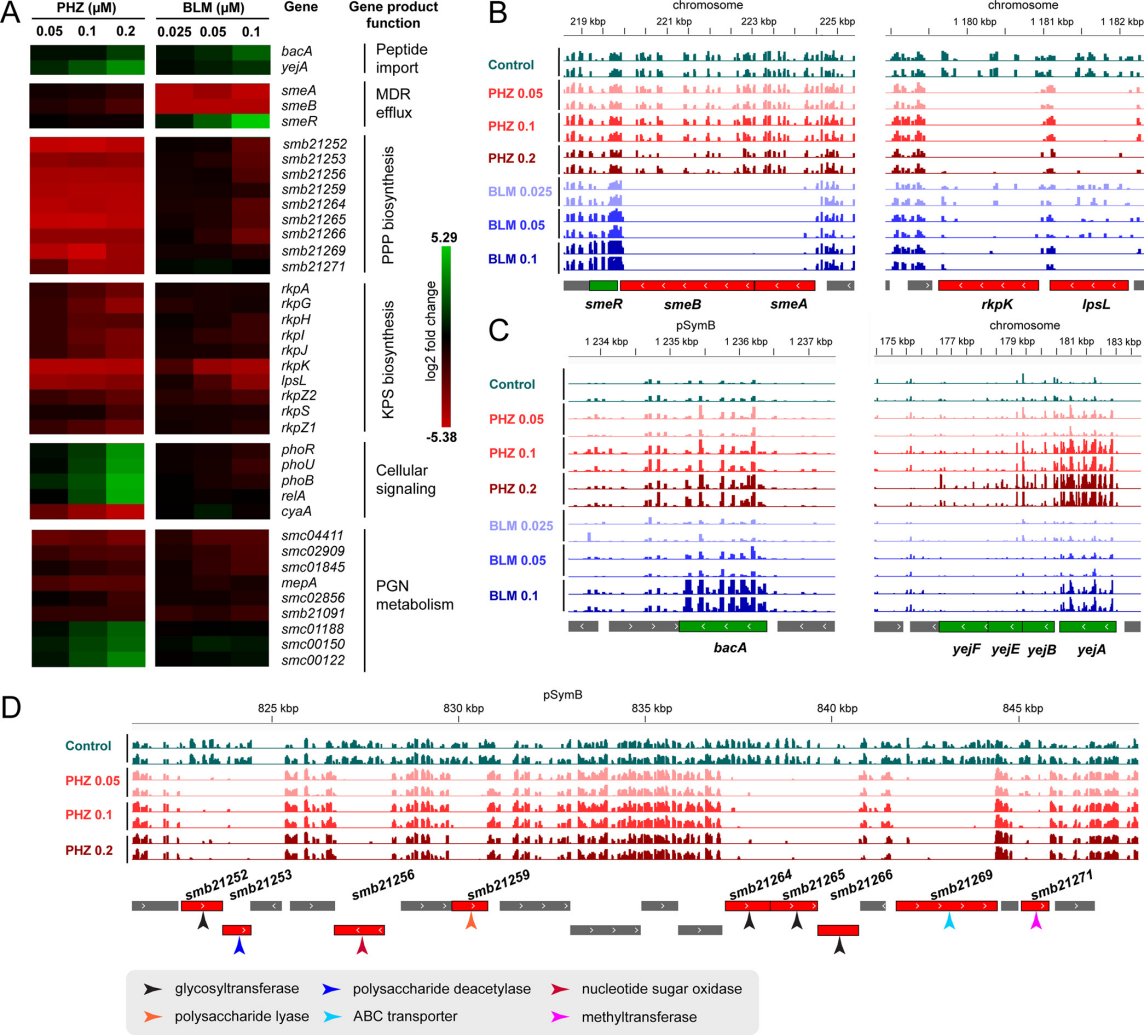
Genetic determinants of S. meliloti PHZ and BLM sensitivity revealed by Tn-seq. (A) Heat map showing the level of depletion or enrichment of transposon insertions in the indicated genes in the S. meliloti population grown in the presence of PHZ or BLM at the indicated concentrations. The color-code scale indicates the log_2_ fold change in the insertion abundance under the test conditions relative to the control conditions. A positive fold change (green) is indicative of genes that are enriched in Tn insertions, and a negative fold change (red) indicates genes with a depleted number of Tn insertions compared to the control conditions. (B to D) IGV (82) view of Tn-seq sequencing data for selected genomic regions of S. meliloti. The histograms indicate the abundance of mutants in the Tn-seq population for the indicated screens. Genes whose products contribute to PHZ or BLM resistance have a lower frequency of Tn insertions in peptide treatment screens than the control, while genes whose products potentiate peptide action harbor more transposon insertions than the control. The genomic regions shown carry the *smeABR* genes, coding for the RND family export pump SmeAB and a cognate transcriptional repressor SmeR, and the *rkpK* and *lpsL* KPS biosynthesis genes (B), the *bacA* and *yejABEF* genes (C), and the major PPP (phazolicin-protecting polysaccharide) biosynthesis gene cluster (D). PGN, peptidoglycan; KPS, capsular polysaccharide; MDR, multidrug resistance.

To identify additional S. meliloti genes that contribute to PHZ sensitivity, we performed a genome-wide transposon sequencing (Tn-seq) screen (44). We expected that the cultivation of a library of S. meliloti mutants containing random single transposon insertions in the presence of PHZ should reveal a decreased frequency of mutants harboring transposons in genes whose products contribute to PHZ resistance. Conversely, mutants harboring transposons in genes whose products potentiate PHZ action should become more frequent. Tn-seq screens with three concentrations below the MIC of PHZ (0.2 μM, 0.1 μM, and 0.05 μM) were performed. As a control, a library grown without PHZ was used. The screens revealed that the transposon insertion frequencies in the absence and presence of PHZ differed significantly for 219 genes (Data Set S1). Screens conducted with lower concentrations of the antibiotic retrieved subsets of genes that were retrieved at higher concentrations (Fig. S5A). The concentration dependence confirms that the changes in the transposon insertion frequencies are indeed due to antibiotic action. As expected, transposon insertions in *bacA* and *yejA* had a positive effect on fitness (increased frequency) (Fig. 5A and C). Of note, *yepA* (*smc00951*), encoding the alternative SBP for the YejBEF permease involved in muropeptide uptake (25), was not affected in the screen, indicating that YepA is not mediating PHZ uptake. Tn-seq did not allow pinpointing a candidate outer membrane uptake system for PHZ since the frequency of insertions in the genes encoding porins and TonB-ExbBD was not affected.

10.1128/mbio.00217-23.5 FIG S5 Tn-seq identification of genetic determinants of PHZ and BLM sensitivity. (A) Venn diagrams indicating the number of genes identified as significantly enriched or depleted in the number of Tn insertions at different concentrations of PHZ and BLM. (B) Heat map of the level of depletion or enrichment of mutants in TolC-dependent transport systems in S. meliloti in the transposon mutant population grown in the presence of PHZ or BLM. The color-code scale indicates the log_2_ fold change in the insertion abundance under the test conditions relative to the control conditions. A positive fold change (green) is indicative of genes that are enriched in Tn insertions, and a negative fold change (red) indicates genes with a depleted number of Tn insertions compared to the control conditions. ABC, ATP-binding cassette transporter; MFP, membrane fusion protein; MFS, major facilitator superfamily transporter; RND, resistance-nodulation-division family transporter; TF, transcription factor. Download FIG S5, JPG file, 2.5 MB. Copyright © 2023 Travin et al. 2023 Travin et al. https://creativecommons.org/licenses/by/4.0/ This content is distributed under the terms of the Creative Commons Attribution 4.0 International license.

10.1128/mbio.00217-23.10 DATA SET S1 TRANSIT analysis of Tn-seq screens with PHZ and BLM. Download Data Set S1, XLSX file, 0.5 MB. Copyright © 2023 Travin et al. 2023 Travin et al. https://creativecommons.org/licenses/by/4.0/ This content is distributed under the terms of the Creative Commons Attribution 4.0 International license.

Our and previous (45) Tn-seq screens revealed few insertions in S. meliloti *tolC*, even for the control grown in the absence of antibiotics, indicating that the loss of the gene has an overall fitness defect and precluding its detection in Tn-seq experiments. TolC functions in conjunction with three types of inner membrane transport systems, ABC transporters, resistance-nodulation-division (RND) family transporters, and major facilitator superfamily (MFS) transporters (9). A comprehensive list of TolC-dependent transport systems in S. meliloti was established previously (46). Our Tn-seq analysis showed that transposon insertions in the genes encoding these transporters do not affect fitness in the presence of PHZ (Fig. S5B).

Tn-seq analysis did not reveal a TolC-dependent efflux system that could secrete PHZ, suggesting that TolC contributes to the PHZ sensitivity of Sm1021 (Table 1) by a mechanism unrelated to PHZ export *per se*. This mechanism could be related to envelope biogenesis since S. meliloti TolC is known to be required for the secretion of several polysaccharides (47). Consistent with this hypothesis, the Tn-seq screens uncovered a role of two surface polysaccharides whose production could depend on TolC in PHZ resistance. Nine genes located between *smb21252* and *smb21271* are necessary for the synthesis of an as-yet-uncharacterized surface polysaccharide (48). Transposon insertions in these genes strongly reduce fitness, even at the lowest tested concentration of PHZ (Fig. 5A and D). We speculate that this polysaccharide, which we provisionally name PPP (PHZ-protecting polysaccharide), forms a protective barrier that either sequesters or repulses PHZ molecules, thereby reducing the effective concentration of the antibiotic in the cell. In A. tumefaciens, several orthologs of the PPP cluster genes were identified in a genetic screen for enhanced sensitivity to the antimicrobial pyocyanin (49), potentially because A. tumefaciens PPP plays an equivalent barrier role for pyocyanin. Moreover, several genes of the S. meliloti PPP cluster are likely involved in legume symbiosis, as indicated by a Tn-seq screen in the host Medicago truncatula (50). A similar barrier role was proposed for diverse surface polysaccharides in the protection of S. meliloti and other bacteria against membrane-disrupting AMPs (51–57). According to Tn-seq, the biosynthetic genes for the S. meliloti capsular polysaccharide KPS also contribute to fitness in the presence of PHZ although to a lesser extent than the PPP biosynthesis genes (Fig. 5A and B).

Several other sets of genes retrieved in the PHZ Tn-seq screen might also be linked to polysaccharide production. For example, transposon insertions in *smc02147*, *smc02141*, and *smc02140*, encoding the phosphate-signaling complex PhoRUB, are enriched in the PHZ Tn-seq screen (Fig. 5A). PhoRUB positively regulates the expression of genes controlling the production of the surface polysaccharides galactoglucan and succinoglycan (58). Transposon insertions in the *relA* gene (*smc02659*), involved in the synthesis of the bacterial second messenger (p)ppGpp, are also strongly enriched in the presence of PHZ (Fig. 5A). The S. meliloti *relA* mutants have pleiotropic phenotypes, including the overproduction of an extracellular polysaccharide (59). Interestingly, while the majority of these phenotypes depend on the RNA polymerase-binding protein DksA, encoded by *smc00469*, polysaccharide synthesis does not (60, 61). Consistently, the Tn-seq data show that *dksA* has no impact on fitness in the presence of PHZ, suggesting that the fitness effect of *relA* mutants in the presence of PHZ is related to extracellular polysaccharide biosynthesis. cAMP is another bacterial secondary messenger reported to regulate surface polysaccharide biosynthesis, including genes that are potentially involved in the synthesis of PPP (62, 63). Notably, the adenylate cyclase gene *cyaA* (*smc00339*) is strongly depleted in our PHZ screen (Fig. 5A). Thus, the identification in our screens of the polysaccharide biosynthetic genes and polysaccharide synthesis regulatory genes *tolC*, *phoRUB*, *relA*, and *cyaA* points to a role of surface polymers in PHZ resistance. While S. meliloti has the genetic potential for the production of a variety of surface polysaccharides (48), we propose that the positively or negatively selected mutations in regulatory genes are involved in PHZ resistance/sensitivity by affecting the production of the PPP and KPS polysaccharides.

Transposon insertions in several genes involved in PGN metabolism also affect the resistance of S. meliloti to PHZ, either negatively or positively (Fig. 5A). These changes in fitness suggest that the PGN layer can modulate the passage of PHZ through the periplasm and that PGN of a specific composition and/or cross-linking state can trap PHZ with different efficiencies.

In addition to the PHZ screen, we performed a screen with 0.1 μM, 0.05 μM, and 0.025 μM BLM as this molecule is also cotransported by BacA and YejABEF. We identified 107 genes in this screen (Fig. S5A and Data Set S1). As for PHZ, insertions in *bacA* and *yejA* had a positive fitness effect with BLM. Interestingly, *bacA* insertions were more enriched in the BLM screens, while *yejA* insertions were more strongly selected by PHZ (Fig. 5A and C), which may indicate uptake preferences. Interestingly, nearly no transposon insertions in the *smc02867* and *smc02868* genes coding for the TolC-dependent AcrAB homolog SmeAB were observed for libraries grown in the presence of BLM. This indicates that contrary to PHZ, TolC expels BLM, in conjunction with the SmeAB efflux system, and that the net sensitivity of S. meliloti to BLM is determined by competition in the periplasm between BacA- and YejABEF-mediated import and SmeAB-TolC-mediated export. Consistently, insertions in the gene encoding SmeR (*smc02866*), a TetR family transcriptional repressor of *smeAB*, led to a strong fitness advantage in the presence of BLM (Fig. 5A and B).

The Tn-seq findings were tested in independently created mutants in eight different genes (Table 1). For each of the eight mutants, the pattern of resistance or sensitivity to PHZ and BLM was consistent with the Tn-seq results. Mutants in the PPP biosynthesis genes *smb21252* and *smb21265* and the KPS biosynthesis gene *rkpK* showed strongly reduced resistance to PHZ, confirming the proposed barrier role of these polysaccharides. In addition, the BLM efflux function of the SmeAB pump was confirmed since the *smeA* mutant was sensitive to this antibiotic at a level similar to that of the *tolC* mutant. For the remaining mutants (*cyaA*, *smc00122* [PGN metabolism], *relA*, and *phoR*), the change relative to the wild-type strain was small, even though the Tn-seq data revealed strong depletion or enrichment of transposon insertions. This shows that Tn-seq has the capacity to reveal subtle phenotypes.

### Conclusions.

**FIG 6:**
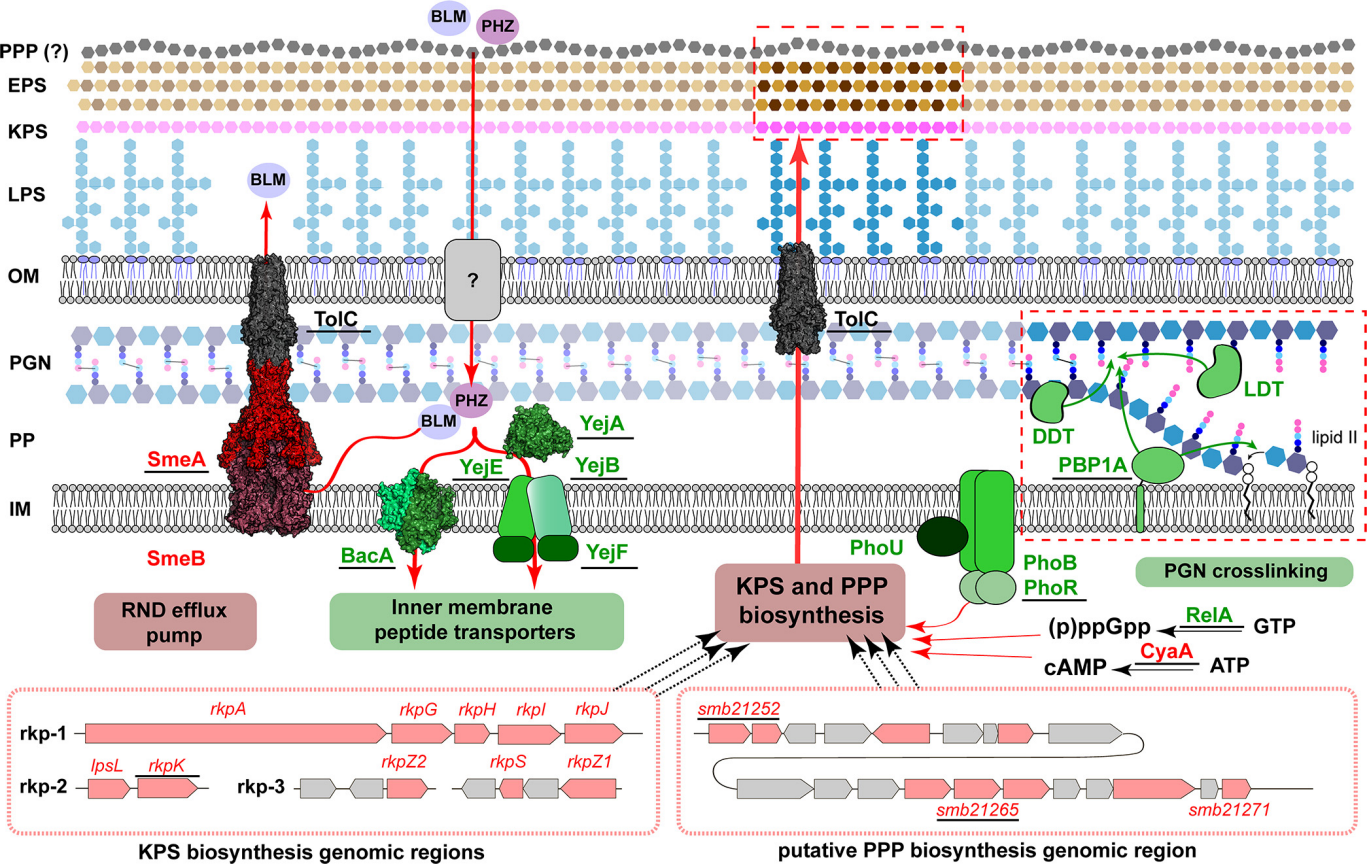
Overview of the major cellular functions affecting the sensitivity of S. meliloti to PHZ and BLM. Functions that contribute to resistance are indicated in red, and those that potentiate the activity of the antimicrobials are highlighted in green. Functions for which the mutants were obtained and tested for sensitivity to PHZ and BLM (Table 1) are underlined. See the text for details. The structure of E. coli AcrAB-TolC (PDB accession no. 5V5S) (88) was used for the representation of the homologous SmeAB-TolC tripartite efflux system. DDT, dd-transpeptidase; EPS, exopolysaccharide; IM, inner membrane; KPS, capsular polysaccharide; LDT, ld-transpeptidase; LPS, lipopolysaccharide; OM, outer membrane; PBP1A, penicillin-binding protein 1A; PGN, peptidoglycan; PP, periplasm; PPP, phazolicin-protecting polysaccharide.

PHZ utilizes a dual-uptake mechanism to cross the inner membrane of S. meliloti Sm1021 via two unrelated peptide transporters, the ABC transporter YejABEF and the SLiPT BacA (Fig. 6). Because these two transporters act independently and are individually sufficient to ensure toxic levels of PHZ inside the cell, both of them need to be inactivated to completely block import. Additionally, diffusion barriers outside the cell as well as in the periplasmic space limit the access of the peptide to inner membrane transporters. The effectiveness of these diffusion barriers is modulated by mutations in the genes involved in the synthesis and/or the regulation of the synthesis of the extracellular polysaccharides KPS and PPP as well as PGN (Fig. 6) but not to an extent where complete resistance can be generated by single mutations in any of these genes. Bacteria that are naturally resistant to PHZ, such as E. coli, may either have very efficient diffusion barriers or rely on alternative mechanisms such as peptide degradation or ribosome rescue (64) that provide PHZ resistance even in the presence of importers capable of its internalization.

The dual-entry mode of PHZ itself dramatically decreases the frequency of spontaneous resistance development (according to our experimental estimation, 1 out of 10^11^ to 10^12^ cells) compared to compounds with a single entry point (1 out of 10^5^ to 10^6^ cells). Moreover, bacteria that managed to acquire PHZ resistance through mutations in both transporters, and even strains with one inactivated transporter, will be unable to develop functional symbioses with legume plants. Since passage through symbiosis and massive multiplication inside the nodules, followed by the return of bacteria to the soil at the end of the nodules’ lifetime, are key mechanisms of rhizobial spread in the environment (65), the loss of any one of these transporters will be evolutionarily disfavored. As such, resistance to PHZ is unlikely to appear, and this compound has clear potential as a biocontrol agent for agriculture, for example, in legume crop inoculation strategies with elite rhizobium strains.

## MATERIALS AND METHODS

### Bacterial strains and growth conditions.

The bacterial strains used in the study are listed in Table S2 in the supplemental material. For S. meliloti, YEB medium (5 g peptone, 5 g beef extract, 5 g sucrose, 1 g yeast extract, and 0.4 g MgSO_4_·7H_2_O [pH 7.5] per L) was used unless specified otherwise. *Rhizobium* sp. Pop5 was cultivated in YM medium (10 g mannitol, 0.5 g K_2_HPO_4_, 0.2 g MgSO_4_, 0.1 g NaCl, and 1 g yeast extract [pH 6.8] per L). E. coli strains were grown in LB medium (5 g NaCl, 10 g tryptone, and 5 g yeast extract per L) or 2× YT medium (5 g NaCl, 16 g tryptone, and 10 g yeast extract per L). Rhizobia were cultivated at 28°C, and E. coli strains were cultivated at 37°C. The following antibiotics were used at the indicated final concentrations: ampicillin (Ap) at 100 μg · mL^−1^, kanamycin (Km) at 50 μg · mL^−1^ for E. coli and 100 μg · mL^−1^ for rhizobia, tetracycline (Tc) at 10 μg · mL^−1^ for E. coli and 5 μg · mL^−1^ for rhizobia, chloramphenicol (Cm) at 34 μg · mL^−1^, spectinomycin (Sp) at 25 μg · mL^−1^, streptomycin (Sm) at 500 μg · mL^−1^, and gentamicin (Gm) at 50 μg · mL^−1^.

10.1128/mbio.00217-23.7 TABLE S2 Bacterial strains and vectors used in the study. Download Table S2, DOCX file, 0.06 MB. Copyright © 2023 Travin et al. 2023 Travin et al. https://creativecommons.org/licenses/by/4.0/ This content is distributed under the terms of the Creative Commons Attribution 4.0 International license.

### Sm1021 transposon library construction.

The S. meliloti Sm1021 strain carrying resistance to Sm was used for transposon mutagenesis and was cultured in YEB medium supplemented with Sm at 28°C. The E. coli MFD*pir* strain (66) (Δ*dapA* derivative, auxotroph for diaminopimelic acid [DAP] synthesis) carrying the plasmid pSAM_Ec (67) was used as a donor strain for transposon mutagenesis and was cultured in LB medium supplemented with 300 μg · mL^−1^ of DAP and Km at 37°C. The donor strain E. coli MFD*pir*/pSAM_Ec and the recipient strain S. meliloti Sm1021 were grown in 50-mL cultures at 180 rpm until the exponential growth phase at a final optical density at 600 nm (OD_600_) of 1. The cultures were washed twice (centrifugation at 1,100 × *g* for 10 min at room temperature) with fresh medium without antibiotics. The pellets were resuspended in fresh medium without antibiotics to obtain a final OD_600_ of 50. For conjugation, the donor strain and the recipient strain were mixed at a ratio of 1:1. Multiple 100-μL drops of the bacterial mix were spotted on YEB agar plates supplemented with 300 μg · mL^−1^ of DAP and incubated at 28°C. After 6 h of incubation, allowing the conjugation of the pSAM_Ec plasmid from the donor E. coli strain to the Sm1021 recipient strain and the transposition of the transposon into the genome of the target strain, the spots were resuspended in YEB medium, and a dilution series was plated onto selective medium carrying Sm and Km and subjected to CFU counting to assess the number of individual bacterial mutants obtained by mutagenesis. In parallel, the remaining bacterial suspension was spread onto YEB agar plates supplemented with Sm and Km to obtain the S. meliloti Sm1021 transposon mutant population. After 2 days of incubation at 28°C, the transposon library was resuspended from the agar plates in fresh liquid YEB medium. The suspension was adjusted to 20% glycerol, aliquoted, and stored at −80°C.

### Selection of phazolicin-resistant mutants.

One hundred microliters of the S. meliloti Sm1021 Tn library prepared as described above with a cell concentration of approximately 1 × 10^8^ cells · mL^−1^ was plated onto two petri dishes with YEB medium containing Sm, Km, and 20 μM PHZ (~20× MIC). Petri dishes were incubated for 48 h at 28°C. The obtained colonies were restreaked onto a petri dish with fresh PHZ-containing medium to confirm the resistance phenotype.

### Whole-genome sequencing and identification of transposon insertion positions.

DNA was extracted from 3-mL cultures of S. meliloti Sm1021 resistant mutants grown overnight using the GeneJET genomic DNA (gDNA) purification kit (Thermo) according to the manufacturer’s protocol. Next-generation sequencing (NGS) libraries were prepared using the NEBNext Ultra II DNA library prep kit (New England BioLabs [NEB]). DNA sequencing was performed on the Illumina MiSeq platform with the 250-bp plus 250-bp paired-end protocol. Library preparation and sequencing were performed at Skoltech Sequencing Core Facilities. Raw reads were filtered and trimmed with Trimmomatic (68), and genome assembly was performed with SPAdes (69). The identification of transposon insertion positions was performed by stand-alone BLAST analysis using the Km^r^ gene sequence as bait (70). The genome annotation under GenBank accession no. NC_003047 was used as a reference.

### Construction of non-PHZ-producing *Rhizobium* sp. strain Pop5 Ω*phzD*.

To obtain a *Rhizobium* sp. Pop5 mutant with a disruption of the *phzD* gene (YcaO domain-containing cyclodehydratase [locus tag RCCGEPOP_21747; GenBank protein accession no. EJZ19165.1]), a 566-bp internal fragment of the gene was PCR amplified and cloned into the plasmid pVO155nptIIgfp (pVO155 plasmid [71] derivative with the constitutively expressed *gfp* gene; does not replicate in *Rhizobium* spp.) between the SalI and XbaI restriction sites. The resulting construct was introduced into *Rhizobium* sp. Pop5 via triparental mating with the helper strain HB101/pRK600 (72). The cells with the plasmid integrated into the genome were selected on YM medium with Km. As there is no resistance marker in the genome of *Rhizobium* sp. Pop5, we did not perform counterselection with the E. coli donor, which could be easily distinguished from *Rhizobium* based on the morphology of the colonies growing on solid YM medium.

### Construction of S. meliloti and E. coli double mutants.

Generalized transduction by S. meliloti Sm1021 phage ϕM12 was used to obtain the double mutants lacking both functional BacA and YejABEF importers. S. meliloti Sm1021 Δ*bacA* served as a donor, while Sm1021 Ω*yejA* and Sm1021 Ω*yejE* were used as recipient strains. The procedure was performed as described previously (73). Briefly, 5 mL of the Sm1021 Δ*bacA* donor strain culture grown overnight at 30°C in LB/MC medium (LB medium with 2.5 mM CaCl_2_ and 2.5 mM MgSO_4_) supplemented with Sp was inoculated by the phage at a cell/phage ratio of 1:1. The mixture was incubated overnight with shaking at 30°C, sterilized by the addition of 150 μL of chloroform, and cleared from the remaining cell debris by centrifugation (7,000 × *g* for 10 min). The obtained lysate was then used to inoculate 1 mL of the culture of the recipient strains grown overnight in LB/MC medium at a cell/phage ratio of 2:1. The obtained mixtures were incubated for 30 min at room temperature and pelleted (4,500 × *g* for 2 min). The pellet was washed with 1 mL of TY medium (5 g tryptone and 3 g yeast extract per L) and resuspended in fresh TY medium. The suspensions were plated onto TY agar plates supplemented with Sp and Km to select for transductants carrying both resistance markers in the genome. A mixture lacking the lysate served as a negative control. The colonies obtained were screened using PCR with primers specific for *bacA* and *yejA* or *yejE* to confirm the genotype.

Gene knockout in E. coli was performed according to a previously described method (74). PCR products used for the transformation of the W3110 strain and the previously constructed W3110 Δ*yejABEF* strain (38) were amplified with the primer pair tolC_F and tolC_R or sbmA_F and sbmA_R from the gDNA of BW25113 *tolC* or *sbmA* single mutant strains from the Keio collection (75). Resistance cassettes were cured with the pCP20 plasmid as described previously (74).

### Molecular cloning procedures.

Table S2 includes the list of plasmids used in the study. Oligonucleotide primers are listed in Table S3. Molecular cloning of the genes encoding BacA-related and YejABEF-related transporters into the pSRK vector (76) was performed either by a conventional restriction enzyme digestion and ligation protocol (restriction sites are specified for each gene in the corresponding primer names) or by a Gibson assembly protocol (NEB) (77). For Gibson assembly, the pSRK plasmid was PCR amplified with primers pSRK_GA_F and pSRK_GA_R and treated with the DpnI restriction endonuclease (Thermo).

10.1128/mbio.00217-23.8 TABLE S3 Nucleotide sequences of primers used in the study. Download Table S3, DOCX file, 0.02 MB. Copyright © 2023 Travin et al. 2023 Travin et al. https://creativecommons.org/licenses/by/4.0/ This content is distributed under the terms of the Creative Commons Attribution 4.0 International license.

The pVO155-nptII-GFP-based vectors containing the fragments of the genes identified by Tn-seq and selected for mutagenesis in Sm1021 were assembled by Gibson assembly (GA) cloning. The plasmid was digested by the XbaI restriction endonuclease. First, for each gene, a PCR fragment of ~600 bp was amplified with gene-specific primers (Phusion DNA polymerase; Thermo) and purified from an agarose gel after electrophoresis (PCR cleanup gel extraction; Macherey-Nagel). This gene fragment served as a template for a second PCR amplification using GA primers (35 nucleotides [nt]) designed in such a way that 20 nucleotides at the 5′ end correspond to the sequence around the XbaI site of the plasmid (Table S3, sequence indicated in red) and the remaining 15 nucleotides correspond to the ends of the first PCR fragment. Amplified fragments of the expected size were purified from the gel and mixed with the digested pVO155 plasmid and Gibson mix (77).

### Tn-seq analysis.

An aliquot of the transposon library of S. meliloti Sm1021 (see above) was diluted in YEB medium to an OD_600_ of 0.01 in 10-mL cultures. PHZ was added in triplicate cultures to a final concentration of 0.2 μM, 0.1 μM, or 0.05 μM. BLM was added in triplicate cultures to final concentrations of 0.1 μM, 0.05 μM, and 0.025 μM. Cultures, including triplicate control cultures without antibiotics, were grown until reaching an OD_600_ of 1 to 3 (6 to 7 generations of growth). Pellets were harvested, and genomic DNA was extracted using the Qiagen QIAamp DNA minikit according to the supplier’s instructions.

Samples of 10 μg of DNA were digested for 1 h at 37°C with 1 μL of the MmeI enzyme (2,000 U · mL^−1^) (catalog no. R0637L; NEB), 25 μL of 10× CutSmart buffer (catalog no. B7204S; NEB), and 10 μL of *S*-adenosine-methionine (1.5 mM) (catalog no. B9003S; NEB) in a total volume of 250 μL. Subsequently, 1 μL of FastAP thermosensitive alkaline phosphatase (1 U · μL^−1^) (catalog no. EF0651; Thermo) was added to the digestion mixes, and samples were incubated for one additional hour at 37°C. The enzymes were then heat inactivated at 75°C for 5 min. Digested DNA samples were purified using the QIAquick PCR purification kit (Qiagen). Seven hundred nanograms of each digested DNA was ligated to experiment-specific barcoded adaptors (5 μM) using T4 DNA ligase (1 U · μL^−1^) (catalog no. EL0016; Thermo) in a final volume of 20 μL and incubated overnight at 16°C. The double-stranded adaptors were generated beforehand by annealing the complementary primers Adaptor-1 and Adaptor-2 (Table S3), as follows: 25 μL of each corresponding single-stranded primer at 200 μM and 1 μL of Tris-HCl (100 μM; pH 8.3) were mixed, the primers in the mixture were denatured at 92°C for 1 min, and the annealing of the complementary primers was promoted by gradual cooling of the samples (2°C per min) in a PCR thermocycler. Transposon borders were subsequently amplified by PCR from the adaptor-ligated DNA samples using 1 μL of them as a template. PCR was performed for 22 cycles using the EuroBio *Taq* polymerase (5 U · μL^−1^) (catalog no. GAETAQ00-4W) in a final volume of 20 μL, according to the manufacturer’s instructions, with 0.5 μM the forward P7 Illumina primer and 0.5 μM the reverse P5 Illumina primer (Table S3). The amplified products were purified by gel extraction on a 2.5% agarose gel using the QIAquick gel extraction kit (Qiagen) and eluted in a final volume of 30 μL. The concentration and the quality control of these Tn-seq samples were assessed using Qubit fluorometric quantification (Thermo) and a Bioanalyzer instrument (Agilent), respectively.

The Tn-seq samples were mixed in equimolar concentrations and sequenced by an Illumina NextSeq 500 instrument with a 2× 75-bp paired-end run at the I2BC sequencing platform (CNRS, Gif-sur-Yvette, France). The generated data were demultiplexed using bcl2fastq2 software (v2.15.0; Illumina) and FASTX-Toolkit (http://hannonlab.cshl.edu/fastx_toolkit/). Only read 1 from each sequenced fragment was used for further analysis. The 3′ transposon sequence was trimmed using Trimmomatic (68), and reads with a length of 75 nucleotides were removed (reads without the transposon insertion). After the trimming step, reads with a length of between 19 and 23 bp were reverse complemented, and only the reads starting with TA dinucleotides were mapped using Bowtie (1.1.2) (78, 79) to the reference genome of S. meliloti (GenBank accession no. NC_003047.1, NC_003078.1, and NC_003037.1). BAM output files were sorted with SAMtools (http://www.htslib.org/). FeatureCounts (80) was used to evaluate the number of reads by gene. BAM output files were converted with SAMtools on the Galaxy server (https://usegalaxy.org/) into nonbinary SAM files, the appropriate format to use for further analysis. The TRANSIT tool (81) with default settings was used for the analysis and identification of genes with fitness defects under the applied selection conditions. The Integrative Genomics Viewer (IGV) tool (82) was used for the visualization of the Tn-seq sequencing data.

### Cocultivation competition experiments and flow cytometry.

For flow cytometry analysis of competition experiments, the GFP-expressing strains Sm1021/pDG71 and Sm1021 Δ*bacA* Ω*yejA* and the DsRed-expressing strains Pop5/pIN72 and Pop5 Ω*phzD*/pIN72 were used. Precultures of the strains, grown in YM medium with the appropriate antibiotics, were washed and diluted in fresh YM medium without antibiotics to an OD_600_ of 0.1. Single-strain cultures or 50%–50% mixtures were prepared from these fresh suspensions to reach a final OD_600_ of 0.4 in YM medium without antibiotics. Aliquots were taken from these cultures at 0 h, 24 h, 48 h, and 88 h for analysis by flow cytometry.

Flow cytometry was performed using a CytoFLEX instrument (Beckman Coulter). Gating on the bacterial particles was done using forward and side scatters, and fluorescence levels of the bacterial particles were acquired using the preset GFP and DsRed channels of the instrument. For each measurement, 50,000 events were recorded and plotted in dot plots in Fig. 2A and Fig. S3A. Data analysis and representation in dot plots were performed with CytExpert version 2.4.0.28 software (Beckman Coulter).

The preparation of mixtures for the cocultivation experiment with CFU number monitoring was performed essentially as described above. wt Sm1021 was used instead of Sm1021/pDG71 to eliminate the effect of plasmid maintenance on the growth of the culture. Aliquots were taken from the cultures at 0 h, 24 h, 48 h, and 72 h. Tenfold dilution series were prepared from these aliquots, and 5 μL of each dilution was spotted onto YM plates without antibiotics or with either Sm or Tc. CFU counting was performed after 48 h of incubation at 28°C.

### Broth microdilution assays and MIC determination.

Precultures of wt S. meliloti Sm1021 and mutants were grown in YEB medium with Sm. Cultures grown overnight were diluted to an OD_600_ of 0.2 in fresh YEB medium with Sm and grown until they reached an OD_600_ of 1. The cells were pelleted by centrifugation and resuspended in YEB medium without antibiotics until an OD_600_ of 0.05 was reached. The cells were dispatched at 150 μL in a 96-well plate, except for the first column, which contained 300 μL of cultures. PHZ was added to the first column to a final concentration of 20 μM, or BLM was added to a final concentration of 13 μM. Twofold serial dilutions in the subsequent columns were obtained by the serial transfer of 150 μL to the next column and mixing by pipetting up and down. No peptide was added to the last column of the 96-well plate. The 96-well plates were incubated in a SPECTROstar Nano plate incubator (BMG Labtech). The growth of the cultures in the wells was monitored by measuring the OD_600_, and data points were collected every hour for 48 h. Plates were incubated at 28°C with double orbital shaking at 200 rpm. Data and growth curves were analyzed using Microsoft Excel. The assay was performed in biological triplicates for both PHZ and BLM.

### CFU assay.

CFU counting was used to assess the sensitivity of strains to the action of PHZ as a complementary method to the broth microdilution assay, as it allows the identification of the occurrence of resistant clones, which appear as colonies growing in the undiluted to hundredfold-diluted samples. For CFU counting, cultures of selected Sm1021 derivatives grown overnight were diluted with fresh YEB medium with relevant antibiotics added to an OD_600_ of 0.2 and allowed to grow to an OD_600_ of 0.6 at 28°C with shaking. Next, the cultures were adjusted to an OD_600_ of 0.2, 10-fold dilution series of the obtained cell suspensions were prepared, and 5 μL of each dilution was spotted onto YEB plates supplemented with either Sm (negative control) or Sm and 8 μM PHZ. Each experiment was performed in triplicate using independent starter cultures inoculated with single colonies of the corresponding strains. For the strains carrying the pSRK plasmids with a panel of *bacA* or *yejABEF* orthologs under the control of the *lac* promoter, 1 mM IPTG was added to the YEB medium to induce expression. Plates were incubated for 3 days at 28°C, after which the number of CFU was counted.

### Production and purification of phazolicin.

Phazolicin was purified from the cultivation medium of *Rhizobium* sp. Pop5 according to a protocol described previously (10). Briefly, the procedure included solid-phase extraction on an Agilent HF Bond Elut LRC-C_18_ cartridge, followed by reverse-phase high-performance liquid chromatography (HPLC) purification on a Luna Prep C_18_ column.

### *In vitro* transport assays with BacA- and SbmA-containing liposomes.

SbmA and BacA proteins for functional assays were purified and reconstituted in small unilamellar vesicles/liposomes using a rapid dilution method as previously described (6). In brief, the proteins were reconstituted in liposomes consisting of POPE [1-hexadecanoyl-2-(9Z-octadecenoyl)-*sn*-glycero-3-phosphoethanolamine]/POPG [1-hexadecanoyl-2-(9Z-octadecenoyl)-sn-glycero-3-phosphoglycerol] lipids at a 1:4 ratio (protein to lipid). The proteoliposomes underwent three rounds of freeze-thawing in liquid nitrogen and were pelleted by ultracentrifugation (100,000 × *g*) for 30 min. The pellet was resuspended in inside buffer (5 mM HEPES-Cl [pH 6.8], 1 mM pyranine [trisodium 8-hydroxypyrene-1,3,6-trisulfonate], 120 mM KCl, and 2 mM MgSO_4_) and subjected to three further rounds of freeze-thawing in liquid nitrogen. The proteoliposomes were harvested by ultracentrifugation (100,000 × *g*) for 30 min, and the pellet was washed to remove any free pyranine and resuspended in a solution containing 5 mM HEPES-Cl (pH 6.8), 120 mM KCl, and 2 mM MgSO_4_. For the transport assay, 15 μM (final concentration) liposomes was placed into a cuvette and quickly mixed with 1.0 mL of outside buffer (5 mM HEPES [pH 6.8], 1.2 mM KCl, and 2 mM MgSO_4_) containing BLM (100 μM) or PHZ (50 μM) with or without valinomycin (1 μM). Data were recorded using a Cary Eclipse fluorescence spectrophotometer (Agilent Technologies) with the following settings: 460-nm excitation, 510-nm emission, 5-nm slit width, and 0.5-s resolution for 6 min.

### Cloning, expression, and purification of YejA^Sm^.

YejA^Sm^ signal peptide prediction was performed using SignalP 5.0 (83)*. yejA*^Sm^ lacking the fragment encoding the first 30 amino acids (signal peptide) was PCR amplified from Sm1021 genomic DNA and cloned into a pET29b(+) vector (Novagen), generating a C-terminal 6×His tag. The resulting plasmid, pET29-*yejA^Sm^*-CHis6, was electroporated into E. coli Rosetta 2(DE3)/pLysS cells. Two liters of 2× YT medium supplemented with Km and Cm was inoculated with 20 mL of a culture of the obtained strain grown overnight. Cells were grown at 37°C at 180 rpm to an OD_600_ of 0.7, induced with 0.5 mM IPTG, and incubated at 28°C for another 5 h. The cultures were cooled on ice, pelleted (4,500 × *g* for 20 min at 4°C), and frozen in liquid nitrogen.

Cells were resuspended in 80 mL of lysis buffer (50 mM Tris-HCl [pH 8.0], 300 mM NaCl, 10% glycerol, 20 mM imidazole) supplemented with homemade purified DNase and a protease inhibitor cocktail (Sigma-Aldrich) and disrupted by sonication. After centrifugation (30,000 × *g* for 25 min at 4°C), the supernatant was loaded onto a 5-mL HisTrap HP column (Cytiva). Protein elution was performed using a solution containing 50 mM Tris-HCl (pH 8.0), 300 mM imidazole, and 300 mM NaCl. Protein fractions were loaded onto a gel filtration column (HiLoad 26/60 Superdex 200 prep grade; Cytiva) equilibrated with a solution containing 50 mM Tris-HCl (pH 8.0) and 150 mM NaCl. The fractions with the highest protein concentrations were pooled, concentrated, and stored at −80°C.

### Crystallization and structure determination of YejA^Sm^.

Crystallization conditions for YejA^Sm^ at 14 mg · mL^−1^ were screened using Qiagen (Valencia, CA) kits with a Mosquito nanodrop robot (SPT Labtech). YejA^Sm^ crystals were manually optimized under the conditions specified in Table S4. Crystals were transferred to a cryoprotectant solution (mother liquor supplemented with 25% polyethylene glycol 400 [PEG 400]) and flash-frozen in liquid nitrogen. Diffraction data were collected at 100 K on the PROXIMA 2 beamline at the SOLEIL synchrotron facility (Saint-Aubin, France). Data processing was performed using the XDS package (84) (Table S4). Because of the diffraction anisotropy, the DEBYE and STARANISO programs developed by Global Phasing Ltd. were applied to the data scaled with AIMLESS using the STARANISO server (http://staraniso.globalphasing.org). These programs perform an anisotropic cutoff of merge intensity data on the basis of an analysis of local *I*/σ(*I*); compute Bayesian estimates of structure amplitudes, taking into account their anisotropic falloff; and apply an anisotropic correction to the data. The structure was solved by molecular replacement with PHASER (85) using the coordinates of the separate N- and C-terminal lobes of the Cu(I)-methanobactin complex-binding protein MbnE from M. parvus OBBP (PDB accession no. 5ICQ [42]) as search models. Inspection of the resulting model using COOT (86) showed strong electron density maps at the interface of the lobes, which were attributed to peptides likely coming from protein degradation during overexpression. The backbones of the short peptide ligands (2- and 5-amino-acid peptides) were modeled at two different places of the interface based on the electron density. Electron density for peptide side chains was more ambiguous, and no electron density linking the two short bound peptides was present, indicating that a population of different peptides might be present in the ligand-binding site of YejA^Sm^ molecules within the crystal. Refinement of the structure was performed with BUSTER-2.10 (87) employing Translation/Libration/Screw (TLS) group restraints. Refinement details are shown in Table S4. Molecular graphic images were generated using PyMOL (http://www.pymol.org).

10.1128/mbio.00217-23.9 TABLE S4 Crystallographic data and refinement parameters. Download Table S4, DOCX file, 0.01 MB. Copyright © 2023 Travin et al. 2023 Travin et al. https://creativecommons.org/licenses/by/4.0/ This content is distributed under the terms of the Creative Commons Attribution 4.0 International license.

### Data availability.

Genome sequencing data for Sm1021 mutants and Tn-seq sequencing data were deposited in the SRA (BioProject accession no. PRJNA760523 and PRJNA888970, respectively). The YejA^Sm^ structure factors and coordinates were deposited in the Protein Data Bank (PDB) (accession no. 7Z8E).
