# Supplementary figures and images for: Dual-Uptake Mode of the Antibiotic Phazolicin Prevents Resistance Acquisition by Gram-Negative Bacteria
Source: mBio. 2023 Feb 21;14(2):e00217-23. doi: 10.1128/mbio.00217-23 (PMC10128002; doi:10.1128/mbio.00217-23)

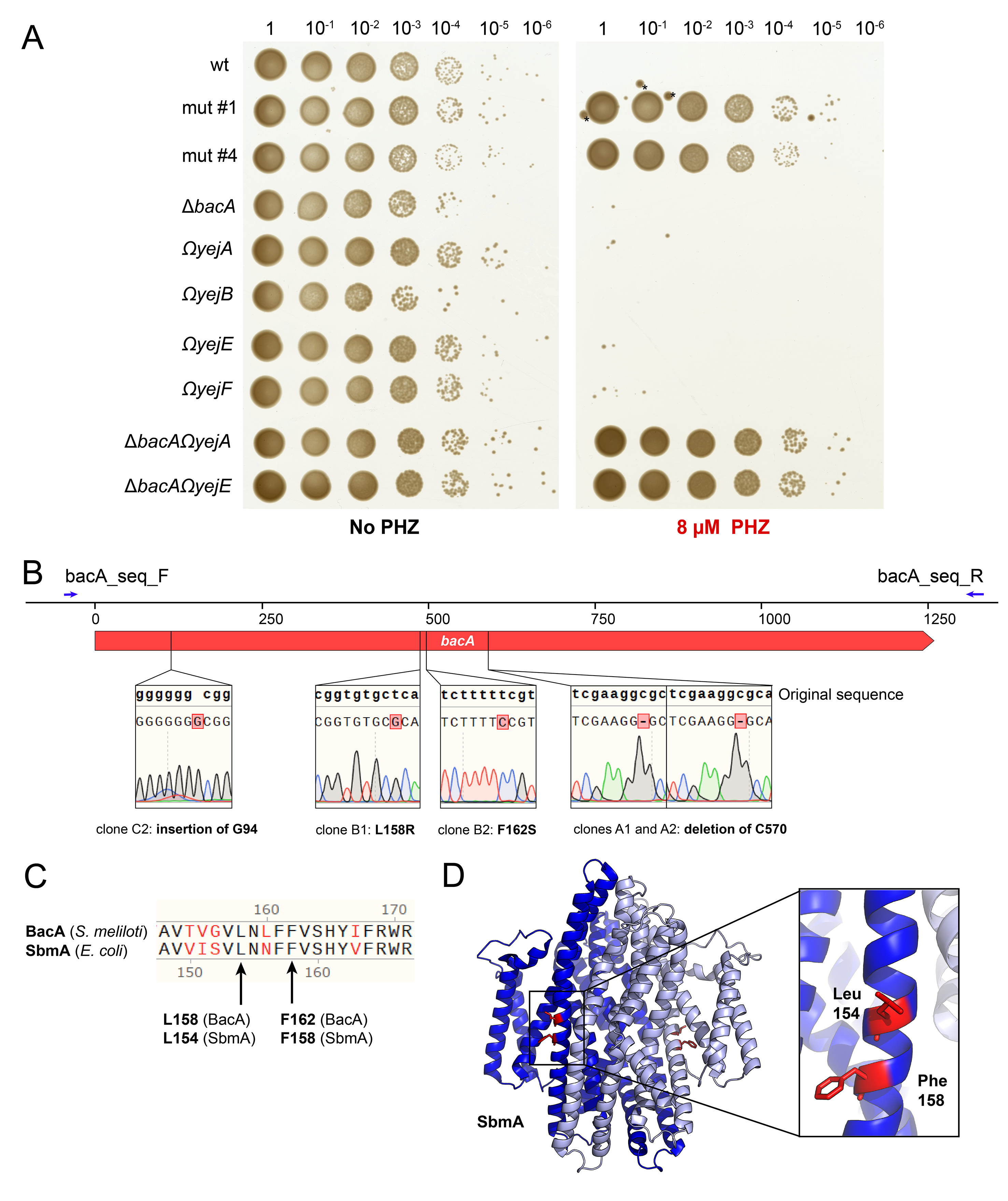

Supplement: FIG S1 [file mbio.00217-23-s0006.jpg]

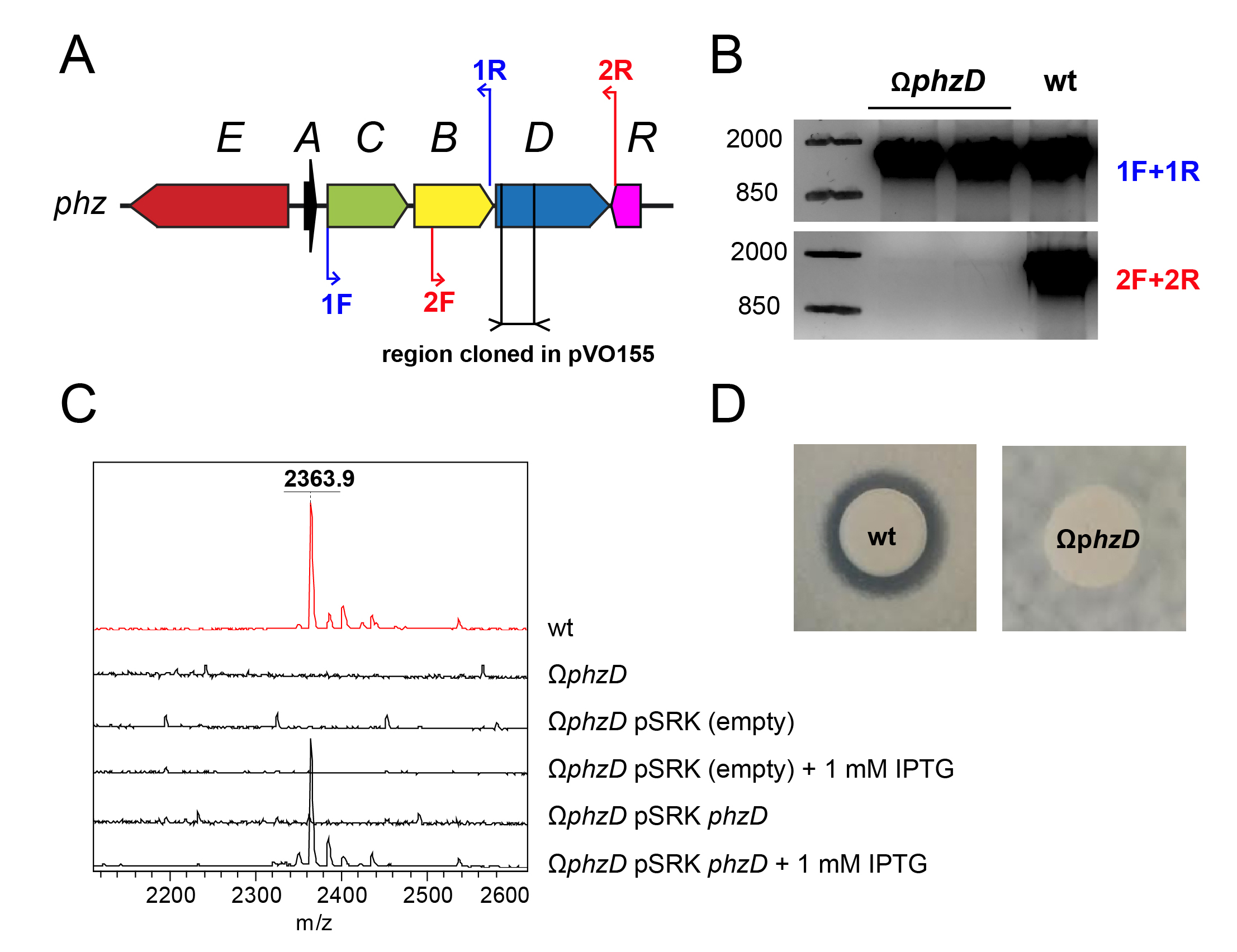

Supplement: FIG S2 [file mbio.00217-23-s0007.jpg]

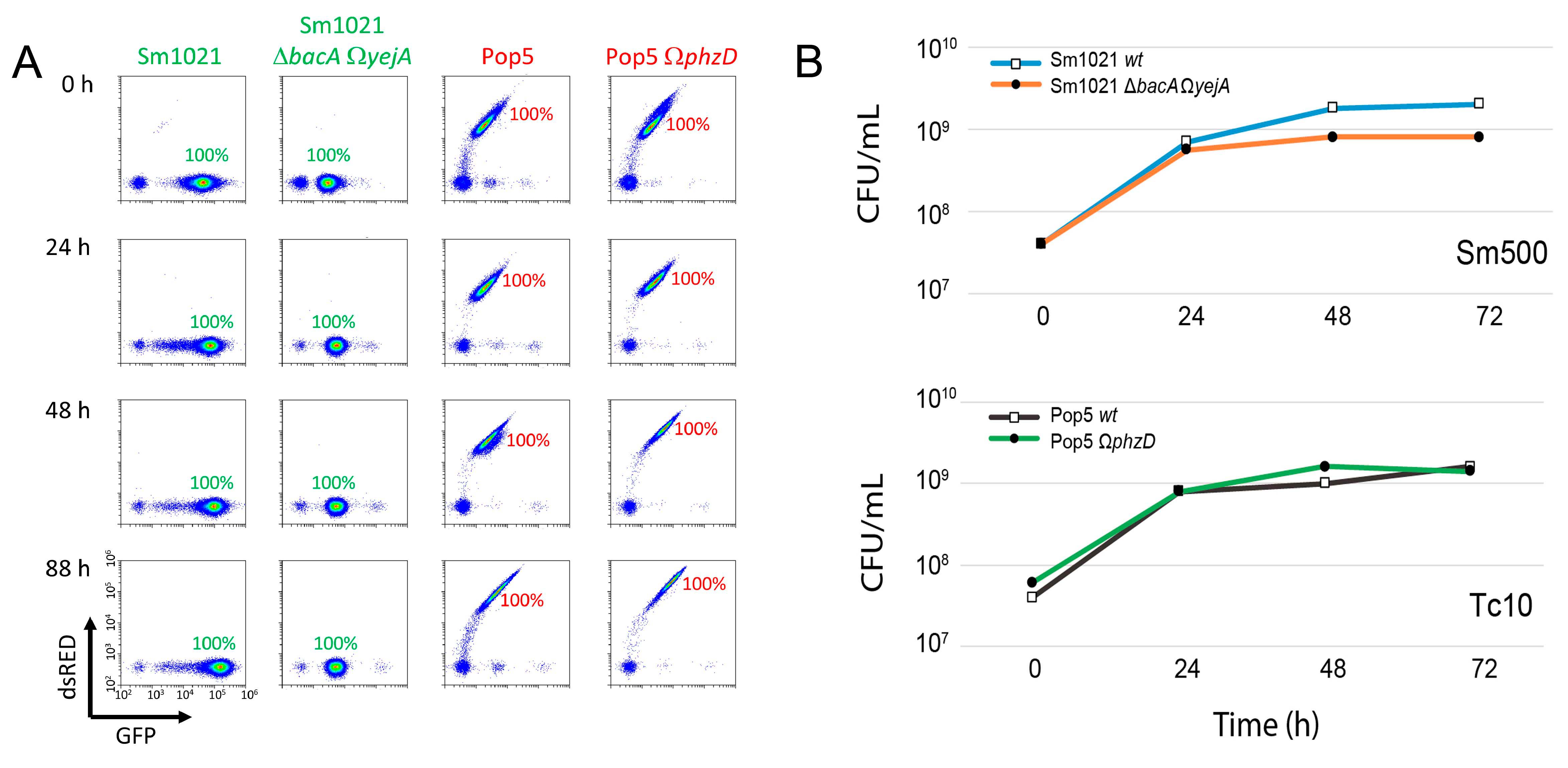

Supplement: FIG S3 [file mbio.00217-23-s0008.jpg]

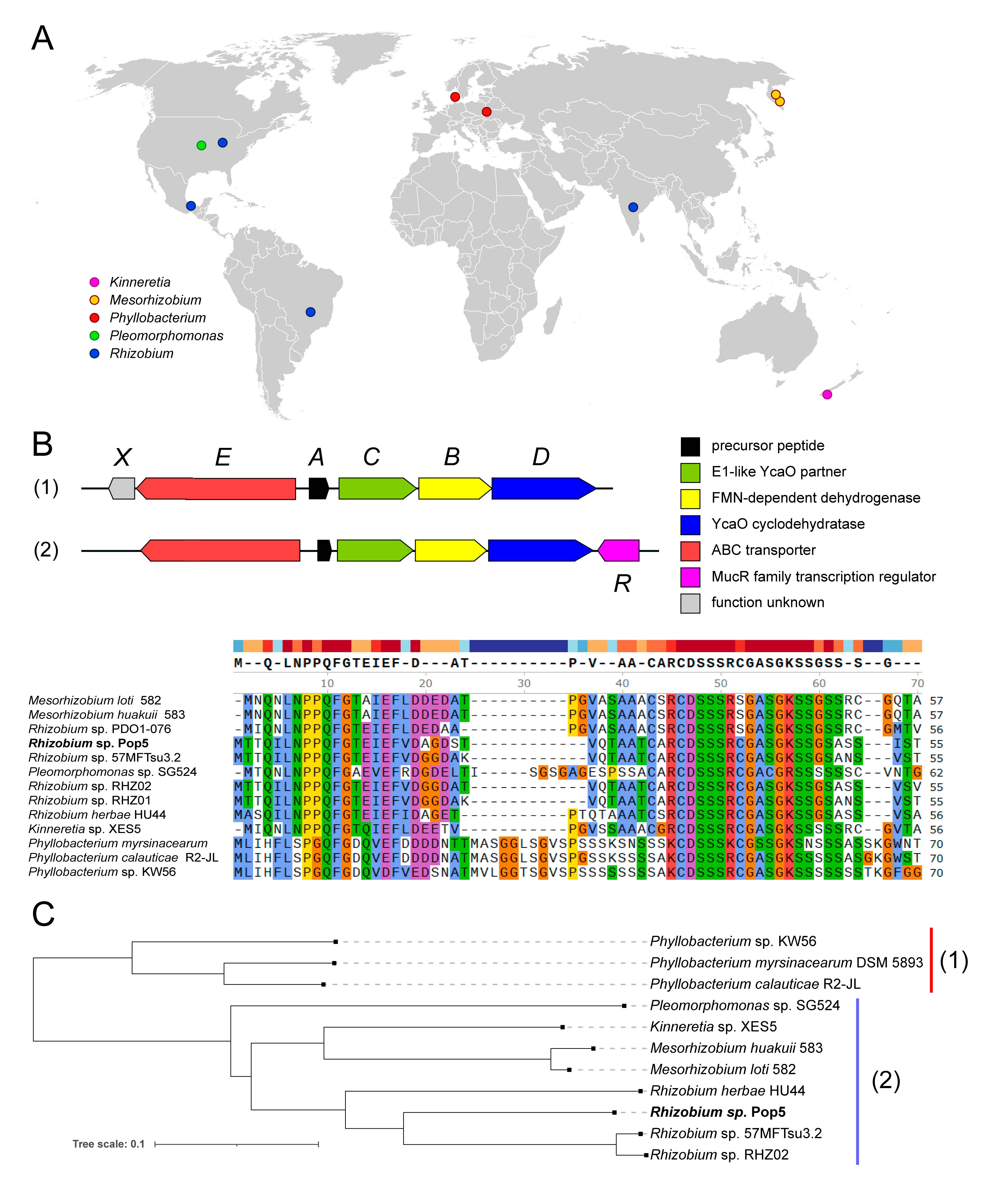

Supplement: FIG S4 [file mbio.00217-23-s0009.jpg]

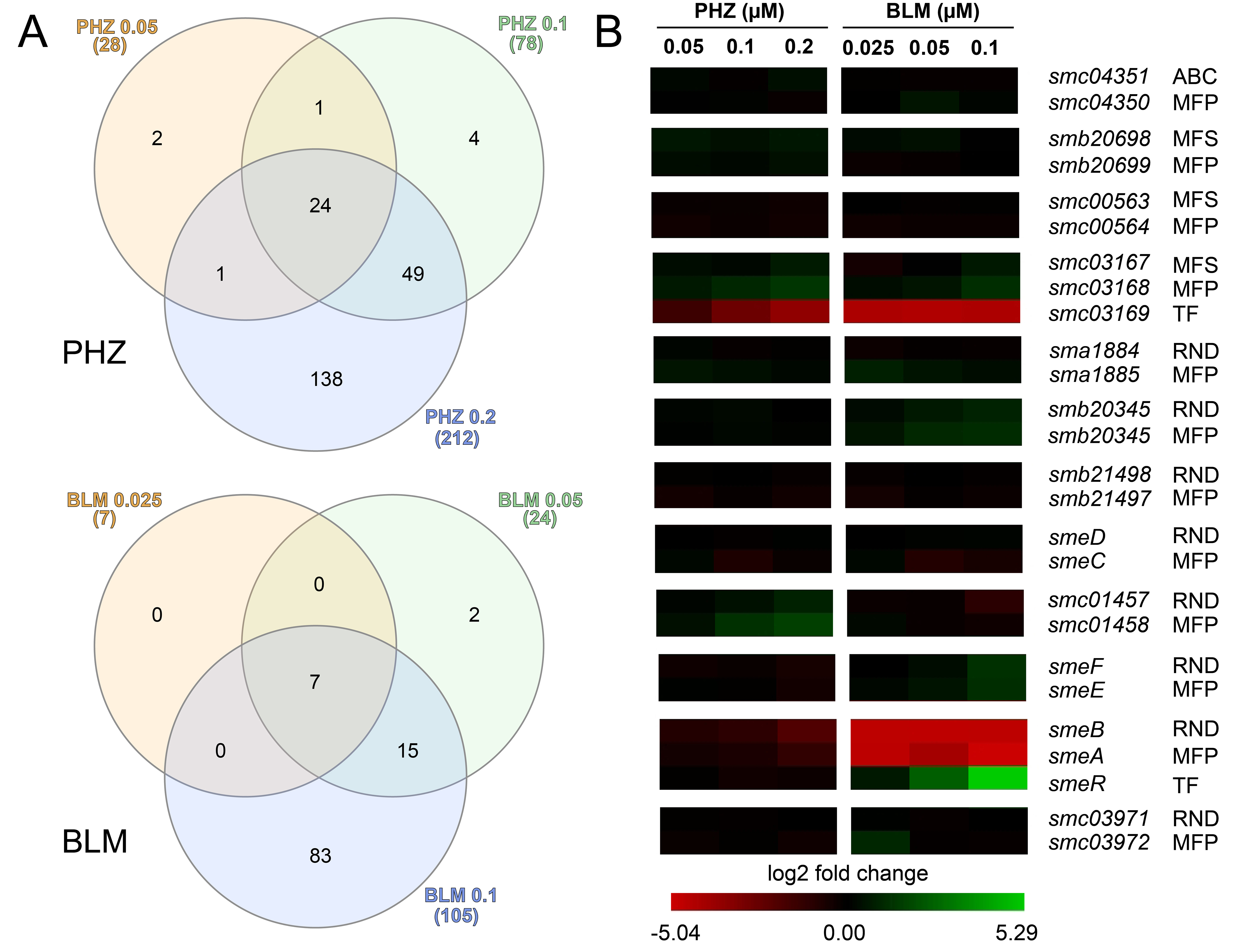

Supplement: FIG S5 [file mbio.00217-23-s0010.jpg]
