# Supplementary material for: Dual-Uptake Mode of the Antibiotic Phazolicin Prevents Resistance Acquisition by Gram-Negative Bacteria
Source: mBio. 2023 Feb 21;14(2):e00217-23. doi: 10.1128/mbio.00217-23 (PMC10128002; doi:10.1128/mbio.00217-23)
Supplement: TABLE S1 [file mbio.00217-23-s0002.docx]

**Supplementary Table 1 | Isolation sources for the strains with *phz*-like BGCs in the genome**

| **Strain** | **Isolation source** | **Accession number and Ref** |
| --- | --- | --- |
| **Kinneretia sp. XES5** | **swab of adult Xenopus laevis skin** | **NZ_CP084752, [1]** |
| **Mesorhizobium huakuii 583** | **Oxytropis kamtschatica root nodules** | **NZ_CP050298, [2]** |
| **Mesorhizobium loti 582** | **Oxytropis kamtschatica root nodules** | **NZ_CP050294, [2]** |
| **Phyllobacterium myrsinacearum DSM 5893** | **NA** | **NZ_SGXB01000013** |
| **Phyllobacterium calauticae R2-JL** | **freshwater sediment** | **NZ_JAGENB010000002, [3]** |
| **Phyllobacterium sp. KW56** | **nodule (species unknown)** | **NZ_JAIQWW010000038** |
| **Pleomorphomonas sp. SG524** | **Sorghum bicolor roots** | **NZ_JAAOYR010000004, [4]** |
| **Rhizobium altiplani BR 10423** | **Mimosa pudica nodules** | **NZ_LNCD01000036, [5]** |
| **Rhizobium herbae HU44** | **Cajanus cajan root** | **NZ_JAEUAO010000003** |
| **Rhizobium sp. Pop5** | **Phaseolus vulgaris root nodule** | **NZ_AMCP01000684** |
| **Rhizobium sp. RHZ01** | **soil** | **NZ_** **JACUZZ010000027** |
| **Rhizobium sp. RHZ02** | **soil** | **NZ_JACUZX010000024** |
| **Rhizobium sp. PDO1-076** | **Populus deltoides root material** | **NZ_AHZC01000156, [6]** |
| **Rhizobium sp. 57MFTsu3.2** | **NA** | **NZ_JDWI01000010** |

**References:**

[1] D. T. Hudson, P. A. Chapman, R. C. Day, X. C. Morgan, and C. W. Beck, “Complete Genome Sequences of Kinneretia sp. Strain XES5, Shinella sp. Strain XGS7, and Vogesella sp. Strain XCS3, Isolated from Xenopus laevis Skin.,” *Microbiol. Resour. Announc.*, vol. 10, no. 50, p. e0105021, Dec. 2021, doi: 10.1128/MRA.01050-21.

[2] V. I. Safronova *et al.*, “Rhizobial microsymbionts of Kamchatka oxytropis species possess genes of the Type III and VI secretion systems, which can affect the development of symbiosis,” *Mol. Plant-Microbe Interact.*, vol. 33, no. 10, pp. 1232–1241, 2020, doi: 10.1094/MPMI-05-20-0114-R.

[3] J. J. M. Lustermans, J. J. Bjerg, A. Schramm, and I. P. G. Marshall, “Phyllobacterium calauticae sp. nov. isolated from a microaerophilic veil transversed by cable bacteria in freshwater sediment.,” *Antonie Van Leeuwenhoek*, vol. 114, no. 11, pp. 1877–1887, Nov. 2021, doi: 10.1007/s10482-021-01647-y.

[4] D. A. Pelletier *et al.*, “Genome Sequences of 42 Bacteria Isolated from Sorghum bicolor Roots.,” *Microbiol. Resour. Announc.*, vol. 9, no. 37, Sep. 2020, doi: 10.1128/MRA.00736-20.

[5] A. C. Baraúna *et al.*, “Rhizobium altiplani sp. nov., isolated from effective nodules on Mimosa pudica growing in untypically alkaline soil in central Brazil,” *Int. J. Syst. Evol. Microbiol.*, vol. 66, no. 10, pp. 4118–4124, Oct. 2016, doi: 10.1099/ijsem.0.001322.

[6] S. D. Brown *et al.*, “Draft genome sequence of Rhizobium sp. strain PDO1-076, a bacterium isolated from Populus deltoides.,” *J. Bacteriol.*, vol. 194, no. 9, pp. 2383–4, May 2012, doi: 10.1128/JB.00198-12.
