# Supplementary material for: Dual-Uptake Mode of the Antibiotic Phazolicin Prevents Resistance Acquisition by Gram-Negative Bacteria
Source: mBio. 2023 Feb 21;14(2):e00217-23. doi: 10.1128/mbio.00217-23 (PMC10128002; doi:10.1128/mbio.00217-23)
Supplement: TABLE S2 [file mbio.00217-23-s0003.docx]

**Supplementary Table 2 | Bacterial strains and vectors used in the study.**

| **Strain** | **Resistance** | **Description** | **Reference or source** |
| --- | --- | --- | --- |
| *S. meliloti* Sm1021 | Sm^R^ | *Sinorhizobium meliloti* wild-type strain | Common laboratory strain |
| *S. meliloti* Sm1021 Δ*bacA* | Sm^R^, Sp^R^ | Sm1021 *bacA654*::Sp (Δ*bacA* null mutant) | [1] |
| *S. meliloti* Sm1021 Ω*yejA* | Sm^R^, Km^R^ | *yejA* plasmid insertion mutant | [2] |
| *S. meliloti* Sm1021 Ω*yejB* | Sm^R^, Km^R^ | *yejB* plasmid insertion mutant | This study |
| *S. meliloti* Sm1021 Ω*yejE* | Sm^R^, Km^R^ | *yejE* plasmid insertion mutant | [2] |
| *S. meliloti* Sm1021 Ω*yejF* | Sm^R^, Km^R^ | *yejF* plasmid insertion mutant | [2] |
| *S. meliloti* Sm1021 Ω*tolC* | Sm^R^, Km^R^ | *tolC* plasmid insertion mutant | [3] |
| *S. meliloti* Sm1021 Δ*bacA* Ω*yejA* | Sm^R^, Km^R^, Sp^R^ | Double mutant in *yejA* and *bacA* obtained through ϕM12 phage transduction | This study |
| *S. meliloti* Sm1021 Δ*bacA* Ω*yejE* | Sm^R^, Km^R^, Sp^R^ | Double mutant in *yejE* and *bacA* obtained through ϕM12 phage transduction | This study |
| *S. meliloti* Sm1021 Ω*smb21252* | Sm^R^, Km^R^ | Insertion mutant in *smb21252* encoding a putative glycosyltransferase proposed to be involved in PPP biosynthesis | This study |
| *S. meliloti* Sm1021 Ω*smb21265* | Sm^R^, Km^R^ | Insertion mutant in *smb21265* encoding a putative glycosyltransferase proposed to be involved in PPP biosynthesis | This study |
| *S. meliloti* Sm1021 Ω*smc02641 (rkpK)* | Sm^R^, Km^R^ | Insertion mutant in *smc02641 (rkpK)* encoding UDP-glucose 6-dehydrogenase involved in capsular polysaccharide biosynthesis [4] | This study |
| *S. meliloti* Sm1021 Ω*smc00122* | Sm^R^, Km^R^ | Insertion mutant in *smc00122* encoding a putative penicillin-binding protein proposed to be involved in PGN biosynthesis | This study |
| *S. meliloti* Sm1021 Ω*smc02147 (phoR)* | Sm^R^, Km^R^ | Insertion mutant in *smc02147 (phoR)* encoding phosphate regulon sensor histidine kinase | This study |
| *S. meliloti* Sm1021 Ω*smc02868 (smeA)* | Sm^R^, Km^R^ | Insertion mutant in *smc02868 (smeA)* encoding membrane fusion protein of SmeAB multidrug-efflux pump [5] | This study |
| *S. meliloti* Sm1021 Ω*smc00339 (cyaA)* | Sm^R^, Km^R^ | Insertion mutant in *smc00339 (cyaA)* encoding adenylate cyclase | This study |
| *S. meliloti* Sm1021 Ω*smc02659 (relA)* | Sm^R^, Km^R^ | Insertion mutant in *smc02659* *(relA)* putative GTP pyrophosphokinase involved in (p)ppGpp biosynthesis | This study |
| *S. meliloti* Sm1021 mut #1 – #4 | Sm^R^, Km^R^ | Phazolicin-resistant mutants selected using transposon library screening | This study |
| *Rhizobium* sp. Pop5 | - | Phazolicin-producing strain (natural isolate) | [6] |
| *Rhizobium* sp. Pop5 Ω*phzD* | Km^R^ | Mutant with pVO plasmid insertion in *phzD* gene, unable to produce mature phazolicin | This study |
| *Rhizobium leguminosarum* 4292 | Rf^R^ | PHZ-sensitive strain | Common laboratory strain |
| *E. coli* Rosetta 2 (DE3) pLysS | Cm^R^ | The strain used for heterologous protein expression | Novagen |
| *E. coli* BL21 (DE3) | - | The strain used for heterologous protein expression | Novagen |
| *E. coli* DH5α | - | The strain used for molecular cloning of all constructs | Common laboratory strain |
| *E. coli* MFDpir Δ*dapA* | - | Donor strain used for transposon library creation, auxotroph for DAP synthesis | [7] |
| *E. coli* W3110 | - | The strain was used for the creation of the triple mutant | Common laboratory strain |
| *E. coli* W3110 Δ*tolC* | - | *tolC* deletion mutant | This study |
| *E. coli* W3110 Δ*yejABEF* | Cm^R^ | *yejABEF* deletion mutant | [8] |
| *E. coli* W3110 Δ*yejABEF* Δ*tolC sbmA::nptII* | Km^R^ | Triple mutant lacking *tolC*, *yejABEF,* and *bacA* | This study |
| **Vector** | **Resistance** | **Description** | **Reference or source** |
| pDG71 | Tc^R^ | Constitutive production of GFP(mut3) under *trp* promoter | [9] |
| pIN72 | Tc^R^ | Constitutive production of the DsRed fluorescent protein | [10] |
| pVO155*npt*II*gfp* | Ap^R^ | Insertional gene inactivation in rhizobia | [11] |
| pET29b(+) | Km^R^ | Expression vector for C-terminally 6His-tagged proteins in *E. coli* | Novagen |
| pET29 *yejA* (Sm) CHis6 | Km^R^ | Expression of C-terminally 6His-tagged YejA^Sm^ in *E. coli* | This study |
| pSAM_Ec | Ap^R^ | Plasmid for *Mariner* transposon library creation | [12] |
| pRK600 | Cm^R^ | Helper plasmid for conjugal DNA transfer | [13] |
| pSRK | Gm^R^ | Broad-host range vector for inducible (*lac* promoter) protein expression, oriV (pBBR5 derivative) | [14] |
| pSRK *phzD* | Gm^R^ | *phzD* from *Rhizobium* sp. Pop5 | This study |
| pSRK *bacA* (Sm) | Gm^R^ | *bacA* from *Sinorhizobium meliloti* Sm1021 | This study |
| pSRK *bclA* (Bsp) | Gm^R^ | *bclA* from *Bradyrhizobium* sp. ORS 285 | This study |
| pSRK *sbmA* (Ec) | Gm^R^ | *sbmA* from *Escherichia coli* MG1655 | This study |
| pSRK *bacA* (Ba) | Gm^R^ | *bacA* from *Brucella abortus* 2308 | This study |
| pSRK *yejABEF* (Sm) | Gm^R^ | *yejABEF* from *Sinorhizobium meliloti* Sm1021 | This study |
| pSRK *yejABEF* (Ec) | Gm^R^ | *yejABEF* from *Escherichia coli* MG1655 | This study |
| pSRK *nppA_1_A_2_BCD* (Pa) | Gm^R^ | *nppA_1_A_2_BCD* from *Pseudomonas aeruginosa* PA14 | This study |

Ap – ampicillin, Cm – chloramphenicol, Gm – gentamycin, Km – kanamycin, Rf – rifampicin, Sm – streptomycin, Sp – spectinomycin, Tc – tetracycline

**References:**

[1] G. P. Ferguson, R. M. Roop, and G. C. Walker, “Deficiency of a Sinorhizobium meliloti BacA mutant in alfalfa symbiosis correlates with alteration of the cell envelope.,” *J. Bacteriol.*, vol. 184, no. 20, pp. 5625–32, Oct. 2002, doi: 10.1128/JB.184.20.5625-5632.2002.

[2] Q. Nicoud *et al.*, “Sinorhizobium meliloti Functions Required for Resistance to Antimicrobial NCR Peptides and Bacteroid Differentiation.,” *MBio*, vol. 12, no. 4, p. e0089521, Jul. 2021, doi: 10.1128/mBio.00895-21.

[3] A. M. Cosme, A. Becker, M. R. Santos, L. A. Sharypova, P. M. Santos, and L. M. Moreira, “The outer membrane protein TolC from Sinorhizobium meliloti affects protein secretion, polysaccharide biosynthesis, antimicrobial resistance, and symbiosis.,” *Mol. Plant. Microbe. Interact.*, vol. 21, no. 7, pp. 947–57, Jul. 2008, doi: 10.1094/MPMI-21-7-0947.

[4] A. Kereszt, E. Kiss, B. L. Reuhs, R. W. Carlson, A. Kondorosi, and P. Putnoky, “Novel rkp gene clusters of Sinorhizobium meliloti involved in capsular polysaccharide production and invasion of the symbiotic nodule: the rkpK gene encodes a UDP-glucose dehydrogenase.,” *J. Bacteriol.*, vol. 180, no. 20, pp. 5426–31, Oct. 1998, doi: 10.1128/JB.180.20.5426-5431.1998.

[5] S. Eda, H. Mitsui, and K. Minamisawa, “Involvement of the smeAB multidrug efflux pump in resistance to plant antimicrobials and contribution to nodulation competitiveness in Sinorhizobium meliloti.,” *Appl. Environ. Microbiol.*, vol. 77, no. 9, pp. 2855–62, May 2011, doi: 10.1128/AEM.02858-10.

[6] D. Y. Travin *et al.*, “Structure of ribosome-bound azole-modified peptide phazolicin rationalizes its species-specific mode of bacterial translation inhibition,” *Nat. Commun.*, vol. 10, no. 1, p. 4563, 2019, doi: 10.1038/s41467-019-12589-5.

[7] L. Ferrières *et al.*, “Silent mischief: bacteriophage Mu insertions contaminate products of Escherichia coli random mutagenesis performed using suicidal transposon delivery plasmids mobilized by broad-host-range RP4 conjugative machinery.,” *J. Bacteriol.*, vol. 192, no. 24, pp. 6418–27, Dec. 2010, doi: 10.1128/JB.00621-10.

[8] D. Pletzer *et al.*, “The Pseudomonas aeruginosa PA14 ABC Transporter NppA1A2BCD Is Required for Uptake of Peptidyl Nucleoside Antibiotics.,” *J. Bacteriol.*, vol. 197, no. 13, pp. 2217–2228, Jul. 2015, doi: 10.1128/JB.00234-15.

[9] D. J. Gage, “Analysis of infection thread development using Gfp- and DsRed-expressing Sinorhizobium meliloti.,” *J. Bacteriol.*, vol. 184, no. 24, pp. 7042–6, Dec. 2002, doi: 10.1128/JB.184.24.7042-7046.2002.

[10] A. C. Vergunst, A. H. Meijer, S. A. Renshaw, and D. O’Callaghan, “Burkholderia cenocepacia creates an intramacrophage replication niche in zebrafish embryos, followed by bacterial dissemination and establishment of systemic infection,” *Infect. Immun.*, vol. 78, no. 4, pp. 1495–1508, 2010, doi: 10.1128/IAI.00743-09.

[11] V. Oke and S. R. Long, “Bacterial genes induced within the nodule during the Rhizobium-legume symbiosis,” *Mol. Microbiol.*, vol. 32, no. 4, pp. 837–849, 1999, doi: 10.1046/j.1365-2958.1999.01402.x.

[12] A. L. Goodman *et al.*, “Identifying genetic determinants needed to establish a human gut symbiont in its habitat.,” *Cell Host Microbe*, vol. 6, no. 3, pp. 279–89, Sep. 2009, doi: 10.1016/j.chom.2009.08.003.

[13] T. M. Finan, B. Kunkel, G. F. De Vos, and E. R. Signer, “Second symbiotic megaplasmid in Rhizobium meliloti carrying exopolysaccharide and thiamine synthesis genes.,” *J. Bacteriol.*, vol. 167, no. 1, pp. 66–72, Jul. 1986, doi: 10.1128/jb.167.1.66-72.1986.

[14] S. R. Khan, J. Gaines, R. M. Roop, and S. K. Farrand, “Broad-Host-Range Expression Vectors with Tightly Regulated Promoters and Their Use To Examine the Influence of TraR and TraM Expression on Ti Plasmid Quorum Sensing,” *Appl. Environ. Microbiol.*, vol. 74, no. 16, pp. 5053–5062, Aug. 2008, doi: 10.1128/AEM.01098-08.
