# Supplementary material for: Dual-Uptake Mode of the Antibiotic Phazolicin Prevents Resistance Acquisition by Gram-Negative Bacteria
Source: mBio. 2023 Feb 21;14(2):e00217-23. doi: 10.1128/mbio.00217-23 (PMC10128002; doi:10.1128/mbio.00217-23)
Supplement: TABLE S3 [file mbio.00217-23-s0004.docx]

**Supplementary Table 3 | Nucleotide sequences of primers used in the study.**

| **Primer name** | **Primer sequence (5’-3’)*^,^**^,^***** | **Purpose** |
| --- | --- | --- |
| bacA_Sm_pSRK_GA_F | ataacaatttcacacaggaaacagcatatgttccaatccttcttcccc | Molecular cloning of *bacA (S. meliloti* Sm1021*)*, *bacA (B. abortus), bclA (Bradyrhizobium* sp. ORS285*)* and *sbmA (E. coli* MG1655*)* genes into the pSRK plasmid by Gibson Assembly (*bacA*^Sm^) or restriction ligation protocol (others). |
| bacA_Sm_pSRK_GA_R | cgaggtcgacggtatcgatattacagagccagctcttcc |  |
| bacA_Ba_NdeI_F | attataCATATGtttgcgtcatttttcccccg |  |
| bacA_Ba_XbaI_R | atattaTCTAGAtcagctcgcccctggttc |  |
| bclA_Bsp_NdeI_F | attattaCATATGaacaatttgcgctcgaccc |  |
| bclA_Bsp_XbaI_R | attataaTCTAGActactcggcgccacccgcc |  |
| sbmA_Ec_NdeI_F | attattaCATATGtttaagtcttttttcccaaagc |  |
| sbmA_Ec_XbaI_R | atattaTCTAGAttagctcaaggtatgggttacttc |  |
| pSRK_GA_F | atgctgtttcctgtgtgaaattg |  |
| pSRK_GA_R | tatcgataccgtcgacctcg |  |
| yejA_Sm_pSRK_GA_F | ataacaatttcacacaggaaacagcataatgccaaacttctgcaggaccg | Molecular cloning of *yejA (S. meliloti* Sm1021*)* gene into the pSRK plasmid by Gibson Assembly. |
| yejA_Sm_pSRK_GA_R | cgaggtcgacggtatcgatatcattttgcagccgtgtttttcg |  |
| yejA_Ec_NdeI_F | taatattaaCATATGattgtgcgcatactgc | Molecular cloning of *yejABEF* genes (*E. coli* MG1655) into the pSRK plasmid. |
| yejF_Ec_XbaI_R | ataattaTCTAGAtcagctcaacgccagtagctg |  |
| yejE_Ec_mut_F | tcatcctgcgtcacatgttgcctaatgccatg | Internal NdeI site elimination from the *yejE*^Ec^ gene. |
| yejE_Ec_mut_R | tggcattaggcaacatgtgacgcaggatgatact |  |
| nppA1_Pa_NdeI_F | taatattaaCATATGcgtcgcctctccttc | Molecular cloning of *nppA_1_A_2_BCD* genes (*P. aeruginosa* PA14) into the pSRK plasmid. |
| nppD_Pa_SacI_R | attataaGAGCTCtcagttttccgcgcttgcc |  |
| yejA_Sm_NoSP_NdeI_F | atttattaCATATGgaggaacaacccgtctggcacc | Molecular cloning of *yejA* gene (*S. meliloti* Sm1021) into the pET29b plasmid. |
| yejA_Sm_XhoI_R | attaattCTCGAGttttgcagccgtgtttttcgacc |  |
| bacA_seq_F | gcatcaggaggcaagtccttg | Amplification of Sm1021 *bacA* gene region for subsequent amplicon Sanger sequencing. |
| bacA_seq_R | gaggcgttgccgattatcgag |  |
| phzC_1F | atgttttcggtttccccgttcgtac | Verification of the pVO155 plasmid insertion into the *phzD* gene. |
| phzB_1R | gcattaattctcctccggataggcaaagg |  |
| phzB_2F | accatcgagtttcccgatg |  |
| phzD_2R | gctctcaagctaaagcaaaataaggc |  |
| phzD_pVO_SalI_F | attatatGTCGACcgagatatcgtgtgacccc | Cloning of the *phzD* gene 541 bp-long fragment into pVO155. |
| phzD_pVO_XbaI_R | attattaTCTAGAcgccaagaccttcgatagc |  |
| phzD_NdeI_F | atattatCATATGcaacggtcatatcgc | Cloning of *phzD* gene into pSRK plasmid. |
| phzD_HindIII_R | gctgttAAGCTTttatgaaaatggcatgggc |  |
| tolC_F | cacgtaacgccaaccttttgcgg | *tolC* knockout in *E. coli* |
| tolC_R | gaagaatgcggcagataacccgt |  |
| sbmA_F | acgctttgtagcggtcatgcg | *sbmA* knockout in *E. coli* |
| sbmA_R | cggttgcggagcctgactac |  |
| Adaptor-1 | ttccctacacgacgctcttccgatctxxxxxnn** | Generation of adaptors for Tn-seq |
| Adaptor-2 | yyyyyagatcggaagagcgtcgtgtagggaaagagt |  |
| Illumina P5 reverse | aatgatacggcgaccaccgagatctacactctttccctacacgacgctcttccgatct | PCR amplification in Tn-seq |
| Illumina P7 forward | caagcagaagacggcatacgagatagaccggggacttatcatccaacctgt |  |
| SMb21252_F | gacctgtggttttgctaccac | Gene fragment amplification, amplification for Gibson Assembly cloning into pVO155 plasmid, and verification of plasmid integration into the *smb21252* gene. |
| SMb21252_R | ctgcgcctccagataggatag |  |
| SMb21252_F_GA | aggactagtggatccctcgagacctgtggttttgc |  |
| SMb21252_R_GA | ggaccatggtcgagatcctcctgcgcctccagata |  |
| G1_ExF | atgaagatcgcctttcatgcgccgctgaaatcg |  |
| G1_ExR | gaagtgtcgcagcgctgccgccagc |  |
| SMb21265_F | gacgtttcacgcaaccgcttc | Gene fragment amplification, amplification for Gibson Assembly cloning into pVO155 plasmid, and verification of plasmid integration into the *smb21265* gene. |
| SMb21265_R | gcggcccaaaacatccttc |  |
| SMb21265_F_GA | aggactagtggatccctcgagacgtttcacgcaac |  |
| SMb21265_R_GA | ggaccatggtcgagatcctcgcggcccaaaacatc |  |
| G2_ExF | gtgtcgccgaaaccgaagatcgccgtc |  |
| G2_ExR | gccggagactgcggtcgatatacaggcaag |  |
| SMc02641_F | ttcaccgtcgtggtgaccaag | Gene fragment amplification, amplification for Gibson Assembly cloning into pVO155 plasmid, and verification of plasmid integration into the *smc02641* gene. |
| SMc02641_R | gtattcggcttgaaggtcagg |  |
| SMc02641_F_GA | aggactagtggatccctcgattcaccgtcgtggtg |  |
| SMc02641_R_GA | ggaccatggtcgagatcctcgtattcggcttgaag |  |
| G3_ExF | atgaaaatcacgatgatcggtgccggctatgtcg |  |
| G3_ExR | cttcatagggatcgcgcgcgcagtccag |  |
| SMc00122_F | gattcgaccgtatggagcgcaggattcc | Gene fragment amplification, amplification for Gibson Assembly cloning into pVO155 plasmid, and verification of plasmid integration into the *smc00122* gene. |
| SMc00122_R | cgctcgatcgtcctctcgttcgagaggaa |  |
| SMc00122_F_GA | aggactagtggatccctcgattgccgagtcggcga |  |
| SMc00122_R_GA | ggaccatggtcgagatcctccgctcgatcgtcctc |  |
| G4_ExF | atgaggcagaaccttcgggacatgaaagccgt |  |
| G4_ExR | ccatataggcgcggtcgagatagaggcgga |  |
| SMc02147_F | ccccaggacagctacctttc | Gene fragment amplification, amplification for Gibson Assembly cloning into pVO155 plasmid, and verification of plasmid integration into the *smc02147* gene. |
| SMc02147_R | gaagacctccaccaattcgtc |  |
| SMc02147_F_GA | aggactagtggatccctcgaccccaggacagctac |  |
| SMc02147_R_GA | ggaccatggtcgagatcctcgaagacctccaccaa |  |
| G5_ExF | ttgaggaacgaggatatcgtgttggatggcgc |  |
| G5_ExR | cggaccgtggtcggtaaccgtcacctc |  |
| SMc02868_F | ccggcaccgtttcaggtcaag | Gene fragment amplification, amplification for Gibson Assembly cloning into pVO155 plasmid, and verification of plasmid integration into the *smc02868* gene. |
| SMc02868_R | gatctggacgcggacatacatg |  |
| SMc02868_F_GA | aggactagtggatccctcgaccggcaccgtttcag |  |
| SMc02868_R_GA | ggaccatggtcgagatcctcgatctggacgcggac |  |
| G6_ExF | atggacattgcgatgcgcatgaaccgaccgatatt |  |
| G6_ExR | caaccttgttgtcggcattcaccacatagacc |  |
| SMc00339_F | cctatgagatggccaccacag | Gene fragment amplification, amplification for Gibson Assembly cloning into pVO155 plasmid, and verification of plasmid integration into the *smc00339* gene. |
| SMc00339_R | cgacgaaacgtcgataggagtg |  |
| SMc00339_F_GA | aggactagtggatccctcgacctatgagatggcca |  |
| SMc00339_R_GA | ggaccatggtcgagatcctccgacgaaacgtcgat |  |
| G7_ExF | gctgcaaagatcggaatccggattcaaggacatc |  |
| G7_ExR | cggaacatagagcgcgaag |  |
| SMc02659_F | accaagatcaagaagctcgac | Gene fragment amplification, amplification for Gibson Assembly cloning into pVO155 plasmid, and verification of plasmid integration into the *smc02659* gene. |
| SMc02659_R | tttcggggtcgagacgtagtcc |  |
| SMc02659_F_GA | aggactagtggatccctcgaaccaagatcaagaag |  |
| SMc02659_R_GA | ggaccatggtcgagatcctctttcggggtcgagac |  |
| G8_ExF | atgatgcgccaatacgagctcgttgagcgcg |  |
| G8_ExR | ccgcgataccgtattcggcgatctcatgcatg |  |
| pVO-F2 | tcctgtcgggtttcgccacctctgacttgagc | Amplification of fragments cloned into pVO155 MCS. |
| pVO-R2 | ggcacagcaattgcccggctttcttgtaacgcg |  |

* Sequences of the restriction sites are capitalized.

** Sequences of overhangs in the primers used for Gibson Assembly cloning are shown in red.

*** xxxxx/yyyyy stands for variable experiment-specific barcode sequences.
