# Supplementary material for: Dual-Uptake Mode of the Antibiotic Phazolicin Prevents Resistance Acquisition by Gram-Negative Bacteria
Source: mBio. 2023 Feb 21;14(2):e00217-23. doi: 10.1128/mbio.00217-23 (PMC10128002; doi:10.1128/mbio.00217-23)
Supplement: TABLE S4 [file mbio.00217-23-s0005.docx]

**Supplementary Table 4 | Crystallographic data and refinement parameters.**

|  | **YejA^Sm^**# |
| --- | --- |
| PDB code | 7Z8E |
| Crystallization conditions | 14% PEG 8K, 0.1 M  Tris-HCl pH 8.5  0.2 M MgCl_2_ |
| Beamline | SOLEIL-PX2 |
| Wavelength (Å) | 0.9793 |
| *Za* | 1 |
| Space group  Cell parameters (Å,°) | *P2_1_2_1_2_1_*  *a*= 59.9  *b*= 73.7  *c*= 140.8 |
| Resolution (Å) | 65.34-1.58  (1.67-1.58) |
| No. of observed reflections | 765515 (40211) |
| No. of unique reflections | 86259 (4780) |
| Completeness spherical (%) | 100 (100) |
| Mean I/σ(I) | 11.3 (0.6) |
| Completeness spherical Staraniso (%) | *85.7 (22.6)* |
| Completeness ellipsoidal Staraniso (%) | *95.5 (59.7)* |
| R_merge_ (%) | *10.2 (164)* |
| R_pim_ (%) | *3.6 (53.8)* |
| Mean I/σ(I) after Staraniso | *13.1 (1.4)* |
| CC_1/2_ | *0.99 (0.54)* |
| *R_cryst_* (%) | 16.9 |
| *R_free_* (%) | 20.2 |
| rms bond deviation (Å) | 0.01 |
| rms angle deviation (°) | 0.93 |
| Average B (Å^2^)  Protein  Peptide 1/2  Solvent | 25  27/29  35 |
| ^a^Clashscore  MolProbity score | 0.83  0.86 |
| ^a^Ramachandran plot (%)  Favoured  Outliers | 98.48  0 |

Values for the highest resolution shell are in parentheses

CC_1/2_ = percentage of correlation between intensities from random hall-dataset

^a^Calculated with MolProbity

Numbers in italic account for statistical values after ellipsoidal mask application by Staraniso.

# A dataset collected from a crystal, which diffracted anisotropically to 1.702 Å along *a**, 1.645 Å along *b** and 1.577 Å along *c**
